# Conversion of monoclonal IgG to dimeric and secretory IgA restores neutralizing ability and prevents infection of Omicron lineages

**DOI:** 10.1073/pnas.2315354120

**Published:** 2024-01-09

**Authors:** Harold Marcotte, Yunlong Cao, Fanglei Zuo, Luca Simonelli, Josè Camilla Sammartino, Mattia Pedotti, Rui Sun, Irene Cassaniti, Marie Hagbom, Antonio Piralla, Jinxuan Yang, Likun Du, Elena Percivalle, Federico Bertoglio, Maren Schubert, Hassan Abolhassani, Natalia Sherina, Concetta Guerra, Stephan Borte, Nima Rezaei, Makiko Kumagai-Braesch, Yintong Xue, Chen Su, Qihong Yan, Ping He, Caroline Grönwall, Lars Klareskog, Luigi Calzolai, Andrea Cavalli, Qiao Wang, Davide F. Robbiani, Michael Hust, Zhengli Shi, Liqiang Feng, Lennart Svensson, Ling Chen, Linlin Bao, Fausto Baldanti, Junyu Xiao, Chuan Qin, Lennart Hammarström, Xinglou Yang, Luca Varani, Xiaoliang Sunney Xie, Qiang Pan-Hammarström

**Affiliations:** ^a^Division of Immunology, Department of Medical Biochemistry and Biophysics, Karolinska Institutet, Stockholm 17165, Sweden; ^b^Changping Laboratory, Beijing 102206, People’s Republic of China; ^c^School of Life Sciences, Biomedical Pioneering Innovation Center, Peking University, Beijing 100871, People’s Republic of China; ^d^Institute for Research in Biomedicine, Università della Svizzera italiana, Bellinzona 6500, Switzerland; ^e^Microbiology and Virology Department, Fondazione Istituto di ricovero e cura a carattere scientifico (IRCCS) Policlinico San Matteo, Pavia 27100, Italy; ^f^Division of Molecular Medicine and Virology, Department of Biomedical and Clinical Sciences, Linköping University, Linköping 58185, Sweden; ^g^Yunnan Key Laboratory of Biodiversity Information, Kunming Institute of Zoology, Chinese Academy of Sciences, Kunming 650023, People’s Republic of China; ^h^Department of Medical Biotechnology, Institute of Biochemistry, Biotechnology and Bioinformatics, Technische Universität Braunschweig, Braunschweig 38106, Germany; ^i^Department of Laboratory Medicine, Hospital St. Georg, Leipzig 04129, Germany; ^j^ImmunoDeficiencyCenter Leipzig, Jeffrey Modell Diagnostic and Research Center for Primary Immunodeficiency Diseases, Hospital St. Georg, Leipzig 04129, Germany; ^k^Research Center for Immunodeficiencies, Pediatrics Center of Excellence, Children’s Medical Center, Tehran University of Medical Sciences, Tehran 14194, Iran; ^l^Division of Transplantation Surgery, Department of Clinical Science, Intervention and Technology, Karolinska Institutet, Stockholm 14186, Sweden; ^m^Department of Immunology, Peking University Health Science Center, Beijing 100191, People’s Republic of China; ^n^State Key Laboratory of Protein and Plant Gene Research, School of Life Sciences, Peking-Tsinghua Center for Life Sciences, Peking University, Beijing 100871, People’s Republic of China; ^o^State Key Laboratory of Respiratory Disease, Guangzhou Institutes of Biomedicine and Health, Chinese Academy of Sciences, Guangzhou 510530, People’s Republic of China; ^p^Division of Rheumatology, Department of Medicine Solna, Center for Molecular Medicine, Karolinska Institutet, Karolinska University Hospital, Stockholm 17176, Sweden; ^q^Rheumatology Unit, Karolinska University Hospital, Stockholm 17176, Sweden; ^r^European Commission, Joint Research Centre, Ispra 21027, Italy; ^s^Key Laboratory of Medical Molecular Virology (Ministry of Education/National Health Commission/Chinese Academy of Medical Sciences), Shanghai Institute of Infectious Disease and Biosecurity, School of Basic Medical Sciences, Fudan University, 200032 Shanghai 200032, People’s Republic of China; ^t^State Key laboratory of Virology, Wuhan Institute of Virology, Chinese Academy of Sciences, Wuhan, Hubei 430071, People’s Republic of China; ^u^Division of Infectious Diseases, Department of Medicine, Karolinska Institute, Stockholm 17177, Sweden; ^v^Guangzhou Laboratory, Guangzhou 510005, People’s Republic of China; ^w^Beijing Key Laboratory for Animal Models of Emerging and Remerging Infectious Diseases, National Health Commission Key Laboratory of Human Disease Comparative Medicine, Institute of Laboratory Animal Science, Chinese Academy of Medical Sciences and Comparative Medicine Center, Peking Union Medical College, Beijing 100021, People’s Republic of China; ^x^National Center of Technology Innovation for Animal Model, Beijing 102206, People’s Republic of China; ^y^Department of Clinical, Surgical, Diagnostic and Paediatric Sciences, University of Pavia, Pavia 27100, Italy

**Keywords:** IgA, SARS-CoV-2, Omicron, antibody engineering, antibody therapy

## Abstract

The emergence of Omicron lineages and descendent subvariants continues to present a severe threat to the effectiveness of vaccines and therapeutic antibodies. We have previously suggested that an insufficient mucosal immunoglobulin A (IgA) response induced by the mRNA vaccines is associated with a surge in breakthrough infections. Here, we further show that the intramuscular mRNA and/or inactivated vaccines cannot sufficiently boost the mucosal secretory IgA response in uninfected individuals, particularly against the Omicron variant. We thus engineered and characterized recombinant monomeric, dimeric, and secretory IgA1 antibodies derived from four neutralizing IgG monoclonal antibodies (mAbs 01A05, rmAb23, DXP-604, and XG014) targeting the receptor-binding domain of the spike protein. Compared to their parental IgG antibodies, dimeric and secretory IgA1 antibodies showed a higher neutralizing activity against different variants of concern (VOCs), in part due to an increased avidity. Importantly, the dimeric or secretory IgA1 form of the DXP-604 antibody significantly outperformed its parental IgG antibody, and neutralized the Omicron lineages BA.1, BA.2, and BA.4/5 with a 25- to 75-fold increase in potency. In human angiotensin converting enzyme 2 (ACE2) transgenic mice, a single intranasal dose of the dimeric IgA DXP-604 conferred prophylactic and therapeutic protection against Omicron BA.5. Thus, dimeric or secretory IgA delivered by nasal administration may potentially be exploited for the treatment and prevention of Omicron infection, thereby providing an alternative tool for combating immune evasion by the current circulating subvariants and, potentially, future VOCs.

## Significance

Considering the high risk of breakthrough infections in individuals with an insufficient mucosal immunoglobulin A (IgA) response, we have engineered various forms of monoclonal IgA antibodies for direct administration to the mucosal surface. The dimerization of IgA, potentially through increased avidity, significantly enhances the potency of broadly neutralizing antibodies tested. Importantly, converting IgG to dimeric and secretory forms of IgA restores neutralizing ability against Omicron variants. When administered intranasally, the dimeric IgA antibody DXP-604 provided both prophylactic and therapeutic protection against Omicron BA.5 in transgenic mice expressing human ACE2. Thus, the nasal spray delivery of dimeric or secretory IgA antibodies holds the potential to effectively block viral infection and enhance mucosal immunity against severe acute respiratory syndrome coronavirus 2.

As severe acute respiratory syndrome coronavirus 2 (SARS-CoV-2) continues to spread worldwide, selection pressure to evade antibodies in convalescent and/or vaccinated individuals has led to viral mutations and the emergence of variants of concerns (VOCs), such as Alpha (B.1.1.7), Beta (B.1.351), Gamma (P.1), and Delta (B.1.617.2) (1). Mutations in the gene encoding the viral spike (S) protein, including the receptor-binding domain (RBD), may lead to a reduced susceptibility to neutralization by antibodies, an increased binding to the angiotensin-converting enzyme 2 (ACE2) receptor on host cells and a higher transmissibility and infectivity (1). The emergence of the Omicron variant in South Africa in November 2021, and its rapid spread worldwide have strengthened concerns about vaccine efficacy and antibody therapy due to the large number of mutations in the S protein (2–4). The original Omicron variant (B.1.1.529) already harbors 37 mutations in the S glycoprotein, including 15 in the RBD (5). This VOC is continuously evolving and has hitherto divided into five major lineages, BA.1 to BA.5 (6). Novel subvariants such as BA.2.75, BQ.1, XBB, EG.5, and BA.2.86, derived from either BA.2 or BA.4/5, have subsequently emerged (https://gisaid.org/hcov19-variants/) (7). The rise of these subvariants seems to stem from their capacity to evade the immune system and infect individuals who are immune to earlier Omicron subvariants (8, 9).

The development of novel antibody therapies that remain efficacious despite virus evolution is thus urgently needed. Widespread reinfections and vaccine breakthrough infections (BTIs) with Omicron have been reported worldwide, and most of the clinically available antibodies are ineffective against this variant (4, 10). Although Omicron causes less severe symptoms than previous VOCs, it still results in a substantial number of hospitalizations and deaths, especially in unvaccinated individuals. Passive antibody preexposure therapy could be beneficial for the protection of individuals at high risk of developing severe diseases, such as immunocompromised patients and elderly individuals, especially in areas/countries with low vaccination/booster rates (11, 12). Recent evidence suggests a shift in the tropism of the Omicron variant towards the upper respiratory tract (13). Viral particles in the upper airways might be more easily released from the nose and mouth, contributing to the increased transmissibility of the Omicron variant (14). The virus might be contained in the upper respiratory tract of individuals who develop a strong local mucosal immune response, resulting in a mild/asymptomatic infection (15). Thus, mucosal immunity may potentially be exploited for therapeutic or prophylactic purposes (16).

Secretory immunoglobulin A (sIgA) is the most abundant Ig type in secretions and is fundamental for mucosal defenses and protection against respiratory viral infections. While serum IgA is predominantly present as a monomer (mIgA), sIgA is composed of two IgA monomers, connected via the joining (J) chain, and associated with the secretory component (SC) (17). Dimeric IgA (dIgA) produced by B cells in the mucosa is translocated across the epithelium via the polymeric Ig receptor (pIgR) (18). On the luminal side of the epithelium, pIgR is cleaved, while a portion, the SC, remains attached, forming sIgA (19). Among the two subclasses of IgA antibodies in humans, IgA1 constitutes a higher proportion in the upper respiratory tract and IgA2 is more abundant in the lower gastrointestinal tract (19, 20). Mucosal IgA dominates the neutralizing antibody response to SARS-CoV-2 in the early phase of infection (21, 22), and dIgA is a more potent neutralizer than IgG against authentic SARS-CoV-2 (23). Thus, delivery of both dIgA and sIgA via nasal spray is potentially the most effective and convenient option to block viral infection and enhance mucosal immunity against SARS-CoV-2.

## Results

### Secretory Anti-RBD IgA Antibodies Are Produced at Low Levels Following Systemic Vaccination.

**Fig. 1.**
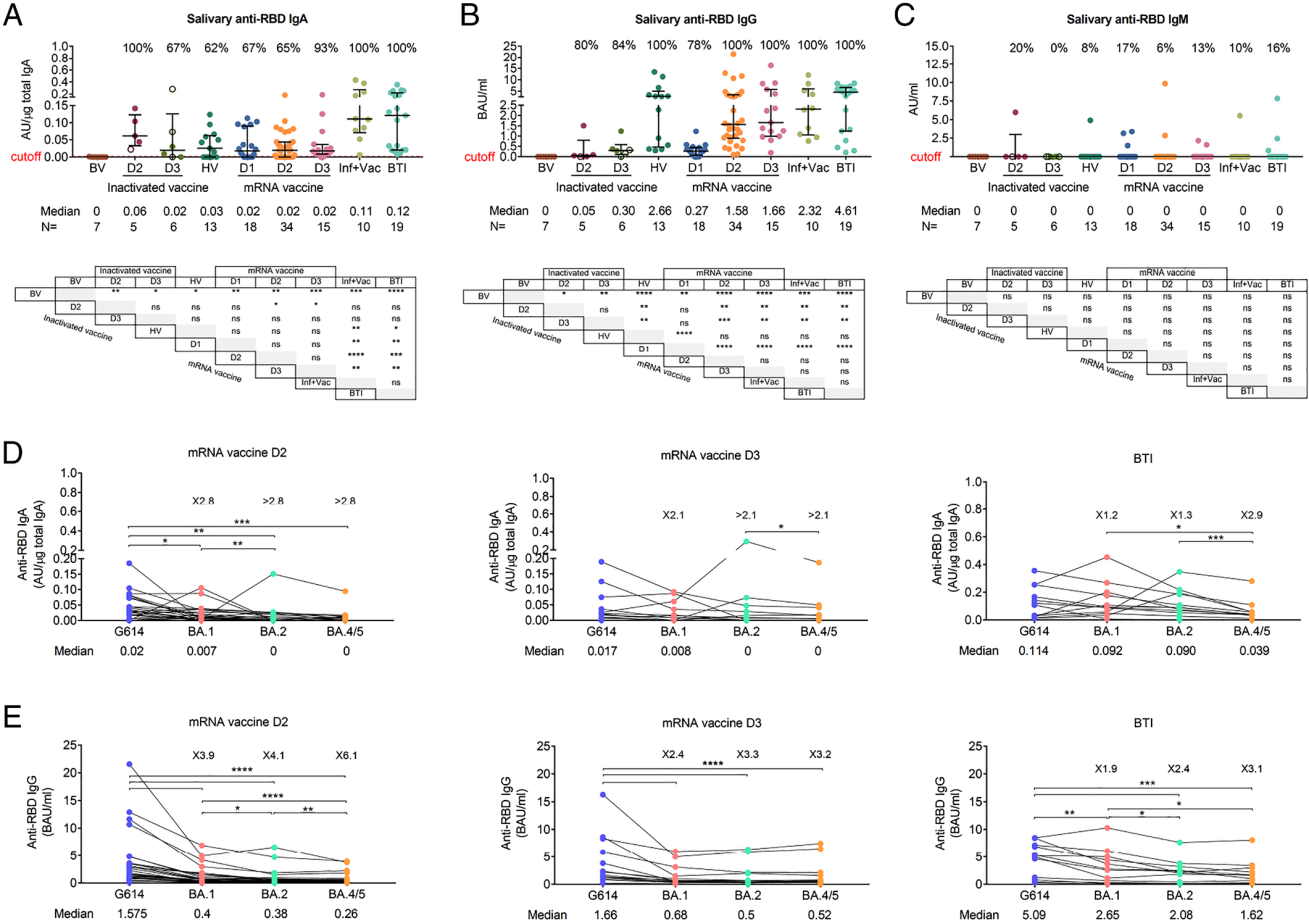
Salivary anti-RBD IgA antibodies are produced at low levels following vaccination. (*A*–*C*) Salivary anti-RBD IgA (*A*), IgG (*B*), and IgM (*C*) antibodies in different vaccination groups. For each group, the number of samples (*n*=) and median antibody titers are shown below the *x*-axis. Whiskers indicate the interquartile range. The results of anti-RBD antibodies are presented as arbitrary units (AU)/µg total IgA (salivary IgA), binding antibody units (BAU/mL) (salivary and plasma IgG) or arbitrary units (AU)/mL (salivary IgM). HV: heterologous vaccination (two doses of inactivated vaccine followed by a heterologous mRNA boost), Inf+Vac: one or two doses of mRNA vaccine after SARS-CoV-2 infection (during the G614 wave), BTI: breakthrough infection (during the BA.1, BA.2, and BA.5 waves) after inactivated and/or mRNA vaccines. A two-sided Mann–Whitney *U* test was used. (*D* and *E*) Salivary anti-RBD IgA (*D*) and IgG (*E*) antibodies against G614 and Omicron variants BA.1, BA.2, and BA.4/5 after the second (D2) and third (D3) doses of mRNA vaccine and following BTI in mRNA-vaccinated individuals. In *A*–*E*, samples were collected 5 to 59 d (median day 20) after each mRNA dose including after mRNA heterologous boost, 6 to 92 d (median day 51) after doses 2 and 3 of inactivated vaccine, and 8 to 43 d (median day 19) after BTI. The number of fold differences of the median compared to G614 are indicated. A Wilcoxon paired-sample signed-rank test was used. *P < 0.05, **P < 0.01, ***P < 0.001, and ****P < 0.0001. ns, not significant. See also *SI Appendix*, Fig. S1.

Natural infections induce a significantly higher level of anti-S and anti-RBD IgA antibodies in saliva, nasal fluid, and bronchoalveolar fluid, compared to vaccinations (21, 24, 25). Among immunoglobulins isotypes, sIgA is present at the highest concentration in saliva and constitutes an accessible marker of the mucosal immune response to SARS-CoV-2 (26, 27). We have previously shown that in individuals receiving mRNA vaccines, higher levels of salivary anti-RBD sIgA antibodies were associated with protection against BTI (28). Here, we further compared the level of salivary antibodies against the RBDs of G614 (wild-type) and of all Omicron lineages in individuals who received various doses of inactivated vaccines or heterologous vaccines (inactivated vaccines + mRNA vaccines) or had confirmed BTI with mild symptoms (Fig. 1 *A*–*C* and *SI Appendix*, Table S1).

The results showed that the median salivary anti-RBD IgA levels in noninfected individuals receiving two or three doses of inactivated whole-virion SARS-CoV-2 vaccine, one to three doses of mRNA vaccine or heterologous vaccination were lower than those after BTI or an mRNA vaccine booster after infection (Fig. 1*A*). Salivary anti-RBD IgG levels after the second and third doses of mRNA vaccine or heterologous mRNA booster dose were similar to those measured after BTI (Fig. 1*B*) while salivary IgM anti-RBD antibodies were detected in less than 20% of individuals after vaccination and BTI (Fig. 1*C*). Lower salivary IgA (from 2.1- to >2.8-fold) and IgG (from 2.4- to 6.1-fold) antibody levels against the RBDs of BA.1, BA.2, and BA.4/5 compared to G614 RBD were observed in vaccinated individuals within 2 mo after two or three doses of mRNA vaccine, but no decrease in antibody levels against Omicron variants was observed in individuals who experienced BTIs during the Omicron BA.1, BA.2, and BA.5 waves, suggesting a long-lasting and broadly cross-reactive mucosal immune response after BTI (Fig. 1 *D* and *E*). Furthermore, a significant increase, more than 10-fold, in salivary IgA and IgG antibodies against Omicron subvariants RBD was observed 2 to 6 wk after BTI (*SI Appendix*, Fig. S1 *A* and *B*).

Levels of salivary anti-RBD IgA antibodies correlated better with levels of RBD-specific secretory Ig (sIg) (R = 0.7879, *P* < 0.0001) than plasma IgA antibodies (R = 0.2822, *P* < 0.0014) (*SI Appendix*, Fig. S1 *C* and *D*), indicating that most of the salivary IgA antibodies measured were produced locally in the salivary glands as sIgA (28). Salivary anti-RBD IgG levels correlated with plasma IgG antibodies, implying that those antibodies were mainly derived from plasma through passive diffusion (R = 0.7098, *P* < 0.0001) (*SI Appendix*, Fig. S1*E*). As previously suggested, our study demonstrated a strong correlation (R = 0.7971, *P* = 0.0006) between the levels of sIgA antibodies in nasal secretions and saliva of individuals who were either vaccinated against SARS-CoV-2 (*n* = 2) or had a BTI (*n* = 13), confirming that salivary sIgA is a reliable marker of mucosal immunity (*SI Appendix*, Fig. S1*F*) (29).

Thus, in uninfected individuals, current vaccine strategies cannot efficiently induce/boost the mucosal IgA response against SARS-CoV-2, especially the Omicron lineages. Considering the high risk of BTI in individuals with insufficient mucosal IgA response (22, 28), our next focus was to develop an effective IgA monoclonal antibody therapy that can be delivered directly at the mucosal surface.

### Characterization of Parental Neutralizing IgG Antibodies.

**Fig. 2.**
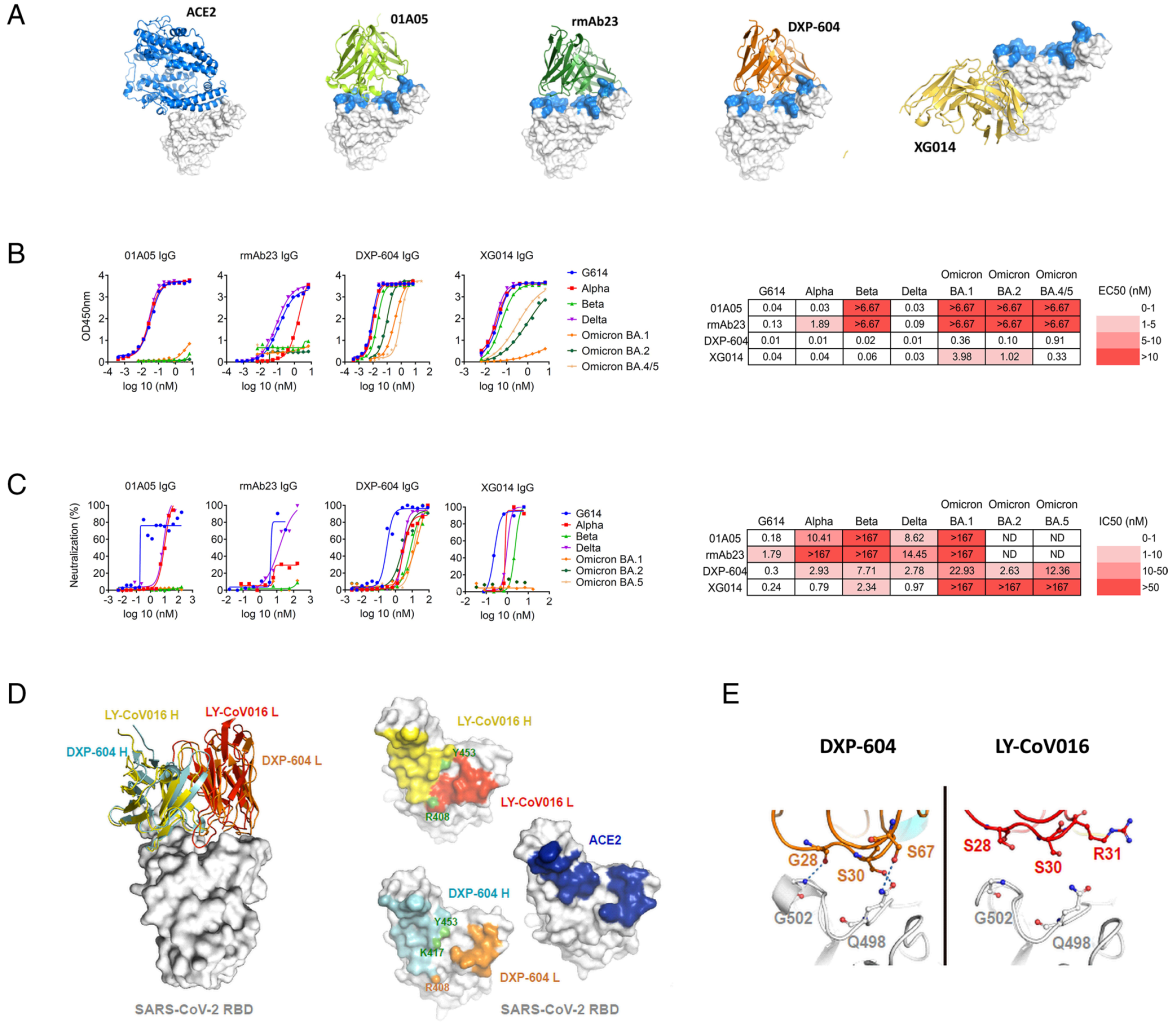
Characterization of neutralizing antibodies 01A05, rmAb23, DXP-604, and XG014. (*A*) In silico binding of ACE2 and IgG antibodies to RBD. The ACE2 receptor binding motif is indicated (blue). (*B* and *C*) Binding to RBD (*B*) and neutralization (*C*) of G614 and VOCs by IgG antibodies. The EC_50_ and IC_50_ and fold-change differences between IgG and IgA antibody forms are indicated. (*D*) Overlaid crystal structures of LY-CoV016 Fab (PDB ID: 7C01) and DXP-604 Fab 473 (PDB ID: 7CH4) in complex with SARS-CoV-2 RBD (*Left* picture) and the footprints of LY-CoV016, DXP-604, and ACE2 (PDB ID: 6M0J) on SARS-CoV-2 RBD. Atoms of the RBD within 5.0 Å of the antibodies or ACE2 are colored yellow (LY-CoV016 H), red (LY-CoV016 L), cyan (DXP-604 H), orange (DXP-604 L), or blue (ACE2) (*Right* picture). (*E*) Hydrogen bonds were formed between S30/S67 in the light chain of DXP-604 and RBD Q498, which is a key ACE2-binding site, and between the main chain groups of G28 and RBD G502. See also *SI Appendix*, Figs. S2–S4.

Four neutralizing IgG mAbs, 01A05 (isolated in this study, *SI Appendix*, *Supporting Text*), rmAb23 (30), DXP-604 (2, 31, 32), and XG014 (33, 34), targeting SARS-CoV-2 RBD, were selected to be converted into IgA formats, based on their distinct binding epitopes and differential cross-neutralizing capacities. All IgG mAbs were originally isolated from peripheral blood mononuclear cells (PBMCs) of convalescent donors who had the wild-type strain (Wuhan or G614 strain) infection (30, 31, 33, 34). Structural analysis indicates that 01A05, rmAb23, and DXP-604 are class I antibodies that bind to the RBD in the up conformation (35), whereas XG014 is a class IV antibody that recognizes a conserved epitope outside the receptor-binding motif in the RBD (33) (Fig. 2*A* and *SI Appendix,* Fig. S2 *A–D*). In summary, the four antibodies recognize two different regions in the RBD, with the Fabs of 01A05 and rmAb23 binding the RBD of the G614 S protein with a lower affinity, having dissociation constants (K_D_) of 2.5 and 6.5 nM (30), respectively, compared to those of DXP-604 and XG014, which show subnanomolar K_D_ (2, 31, 33, 34).

All four antibodies bound RBDs from G614, Alpha and Delta with one-half maximal effective concentration (EC_50_) values from 0.01 to 1.89 nM (Fig. 2*B*) but only DXP-604 and XG014 bound to RBDs from Beta, and Omicron BA.1, BA.2, BA.4/5 (EC_50_: 0.02 to 9.48 nM) (Fig. 2*B*). In accordance with the ELISA results, 01A05 neutralized only G614, Alpha and Delta (half maximal inhibitory concentration (IC_50_): 0.18, 10.41, and 8.62 nM, respectively), rmAb23 neutralized only G614 and Delta (IC_50_: 4.44 and 14.45 nM) while XG014 efficiently neutralized G614, Alpha, Beta, and Delta (IC_50_: 0.24 to 2.34 nM) but poorly neutralized Omicron. DXP-604 neutralized G614 (IC_50_: 0.3 nM) and all VOCs tested (IC_50_: 2.63 to 22.93 nM), although it was less effective against Omicron BA.1 and BA.5 (Fig. 2*C*).

Computational structure modeling was performed based on the binding of 01A05 to the RBD of variants Alpha, Beta, Delta, and Omicron or through previously published information describing docking (rmAb23) (30) or crystallization studies [Protein Data Bank (PDB) ID: 7CH4 for DXP-604 and PDB ID: 7V2A for XG014] (*SI Appendix*, Fig. S3 *A*–*D*). The binding and neutralization assay results were in accordance with structural models. However, mutations in RBD residues within the binding epitope of DXP-604 resulted in a less pronounced decrease in neutralization activity (36). The apparent resistance of DXP-604 to SARS-CoV-2 mutations was confirmed in a S-pseudotype vesicular stomatitis virus neutralization assay showing its potent neutralizing (IC_50_: 0.01 to 1.6 nM) effect against 15 known SARS-CoV-2 variants and other clade 1b sarbecoviruses circulating among other species, including RaTG13 and Pangolin-GD (37) (*SI Appendix,* Fig. S4).

Further analysis of the X-ray crystal structure (31) revealed that the footprint of the DXP-604 heavy chain on the RBD is similar to that of LY-CoV016 (etesevimab) but a higher degree of overlap exists between that of the DXP-604 light chain and ACE2-contact surface (Fig. 2 *D* and *E*) (2). Thus, in contrast to 01A05, rmAb23, and other class I antibodies, the high binding affinity and ACE2-mimicking epitope enabled DXP-604 to exhibit a higher tolerance for RBD substitutions and to retain a broad neutralization activity.

### Conversion of Monoclonal IgG to IgA1 Antibodies Increases the Neutralization Potency.

**Fig. 3.**
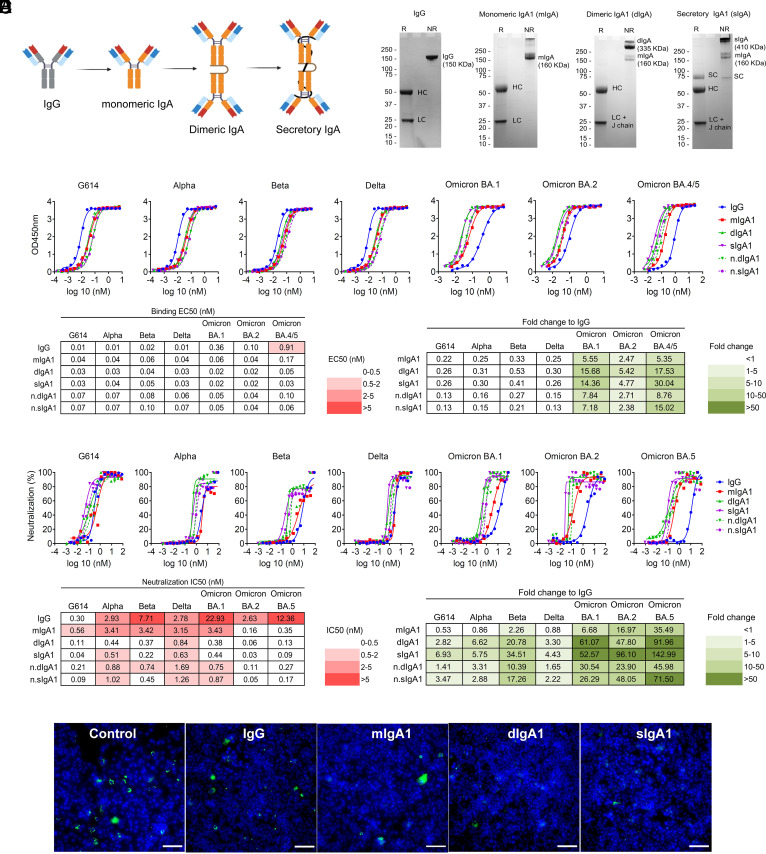
Dimeric and secretory IgA1 showed enhanced binding and neutralization activity against VOCs. (*A*) Illustration showing antibodies engineered from IgG into monomeric, dimeric, and secretory IgA1. (*B*) SDS–PAGE under reducing (R) and nonreducing (NR) conditions showing the assembly and purity of DXP-604 IgG and IgA1 antibodies. HC, heavy chain; LC, light chain; SC, secretory component; J chain, joining chain. The J chain migrates at the same molecular weight as the light chain. (*C* and *D*) Binding to RBD (*C*) and neutralization (*D*) of G614 and VOCs by DXP-604 IgG and IgA [monomeric (mIgA1), dimeric (dIgA1), and secretory IgA1 (sIgA1)] antibodies. The EC_50_ and IC_50_ and fold-change differences between IgG and IgA1 antibody forms are indicated. n.dIgA1 and n.sIgA1 represent normalized values according to the number of binding sites. (*E*) Staining of virus following infection of Vero E6 cells with SARS-CoV-2 Omicron BA.1 preincubated with 3.3 nM DXP-604 IgG or IgA1 forms. Omicron BA.1-infected cells were used as a negative control (control). SARS-CoV-2 virus was visualized using Alexa 488 (green)-conjugated antibody, and the nucleus was stained with DAPI (blue). (Scale bar, 100 μM.) See also *SI Appendix*, Figs. S5 and S6.

The four monoclonal IgG antibodies were subsequently engineered as monomeric (mIgA1), dimeric (dIgA1, via coexpression of the J chain), and secretory (sIgA1, by coexpression of the J chain and SC) IgA1 antibodies to compare the binding and neutralizing properties of the various forms of IgA1 (Fig. 3 *A* and *B*). The binding (EC_50_) and neutralization (IC_50_) values were compared between monomers and dimers after normalization for the number of antibody binding sites.

The conversion of IgG to mIgA1 did not strongly increase the binding affinity of the antibodies for the RBD, with generally less than a twofold increase as measured by ELISA (Fig. 3*D* and *SI Appendix*, Fig. S6). However, dimerization of IgA1 greatly increased the binding of DXP-604 antibodies to the RBD of Omicron BA.1, BA.2, and BA.4/5 (EC_50_: 0.04 to 0.10 nM) from 2.4- to 15.0-fold compared to those of the parental IgG antibodies (Fig. 3*C*). In addition, XG014 dIgA1 and sIgA1 bound to the BA.2 and BA.4/5 RBDs (EC_50_: 0. 14 to 0. 26 nM) 2.4- to 5.0-fold more efficiently than the parental IgG antibodies (*SI Appendix*, Fig. S5*C*).

Switching from IgG to IgA1 and dimerization further increased antibody neutralization activity against real virus, G614 and VOCs, and the effect was greatest for DXP-604, 01A05, and rmAb23 (Fig. 3*D* and *SI Appendix*, Fig. S6). DXP-604 mIgA1 showed increased neutralization activity against BA.1, BA.2, and BA.5 by 6.7- to 35.5-fold (IC_50_: 0.16 to 3.43 nM) (Fig. 3*D*). More importantly, DXP-604 dIgA1 and sIgA1 showed increased neutralization activity against all variants, but particularly against the Omicron lineages BA.1, BA.2, and BA.5, which was 23.9- to 71.5-fold higher than the parental IgG (Fig. 3 *D* and *E*). DXP-604 dIgA1 and sIgA1 neutralized Omicron BA.1, BA.2, and BA.5 (IC_50_: 0.05 to 0.75 nM) to a level that is similar to the counterpart IgG antibodies against G614 (IC_50_: 0.30 nM) (Fig. 3*D*).

An increase in neutralization activity by mIgA1 compared to parental IgG was also observed for 01A05 against G614 (IC_50_: 0.04 nM, by 4.4-fold) and Alpha (IC_50_: 0.93 nM, by 11.2-fold), and for rmAb23 against G614, Alpha and Delta (IC50: 0.89 to 3.42 nM, by 4.2- to >100.6-fold) (*SI Appendix*, Fig. S6 *A* and *B*). Dimerization also improved the neutralizing activity of 01A05 and rmAb23 against G614, Alpha and Delta by 2.3- to >89-fold compared to IgG (*SI Appendix*, Fig. S6 *A* and *B*). Interestingly, switching to IgA1 and dimerization rescued the neutralizing activity of rmAb23 against Alpha and XG014 against Omicron BA.1, to some extent (IC_50_: 1.87 to 8.89 nM for dIgA1 and sIgA1) (*SI Appendix*, Fig. S6 *B* and *C*). These results suggest that the conversion of IgG to IgA1, particularly to the dimeric and secretory forms of IgA1, significantly improved the neutralizing potency of the antibodies against various VOCs.

### Increased Neutralizing Potency of Dimeric IgA1 Is Associated with Increased Avidity.

Enhanced neutralization of dimeric IgA1 antibodies exhibited different patterns, suggesting that the epitope and affinity, in addition to valency, may affect their potency (38). The increase in neutralization potency was more profound against variants for which the parental IgG showed lower (but presence of) neutralizing activity. For instance, DXP-604, rmAb23, and XG014 IgG exhibited significant potency against G614, with IC_50_ values ranging from 0.18 to 0.30 nM, and their neutralization activity did not show a substantial increase upon IgA dimerization. In contrast, the rmAb23 IgG antibody displayed low neutralization activity against G614 (IC_50_: 4.4 nM), but this activity markedly increased by 15- and 27-fold when it was converted into dIgA1 or sIgA1, respectively (*SI Appendix*, Fig. S6*B*). Furthermore, the fold-change improvement in binding and neutralizing activity of the IgA1 forms was positively correlated with the EC_50_ values of the parental IgG (*SI Appendix*, Fig. S7 *A*–*D*) (23). For example, DXP-604, which showed a low RBD binding (EC_50_: 0.91 nM) and a low neutralizing activity (IC_50_: 12.36 nM) against BA.5 as an IgG, was found to be 8.8-fold more potent in binding the RBD and 46.0-fold more potent in neutralizing BA.5 as a dIgA1 (*SI Appendix*, Figs. S3 *C* and *D* and S7 *B* and *D*). In addition, for DXP-604, the fold-change increase in RBD binding correlated with the fold-change increase in neutralizing activity (*SI Appendix*, Fig. S7 *E* and *F*). An increase in neutralizing activity may thus be associated with an increased binding to the RBD, at least for this antibody, for which a correlation analysis could be individually performed due to its broad neutralizing capacity.

**Fig. 4.**
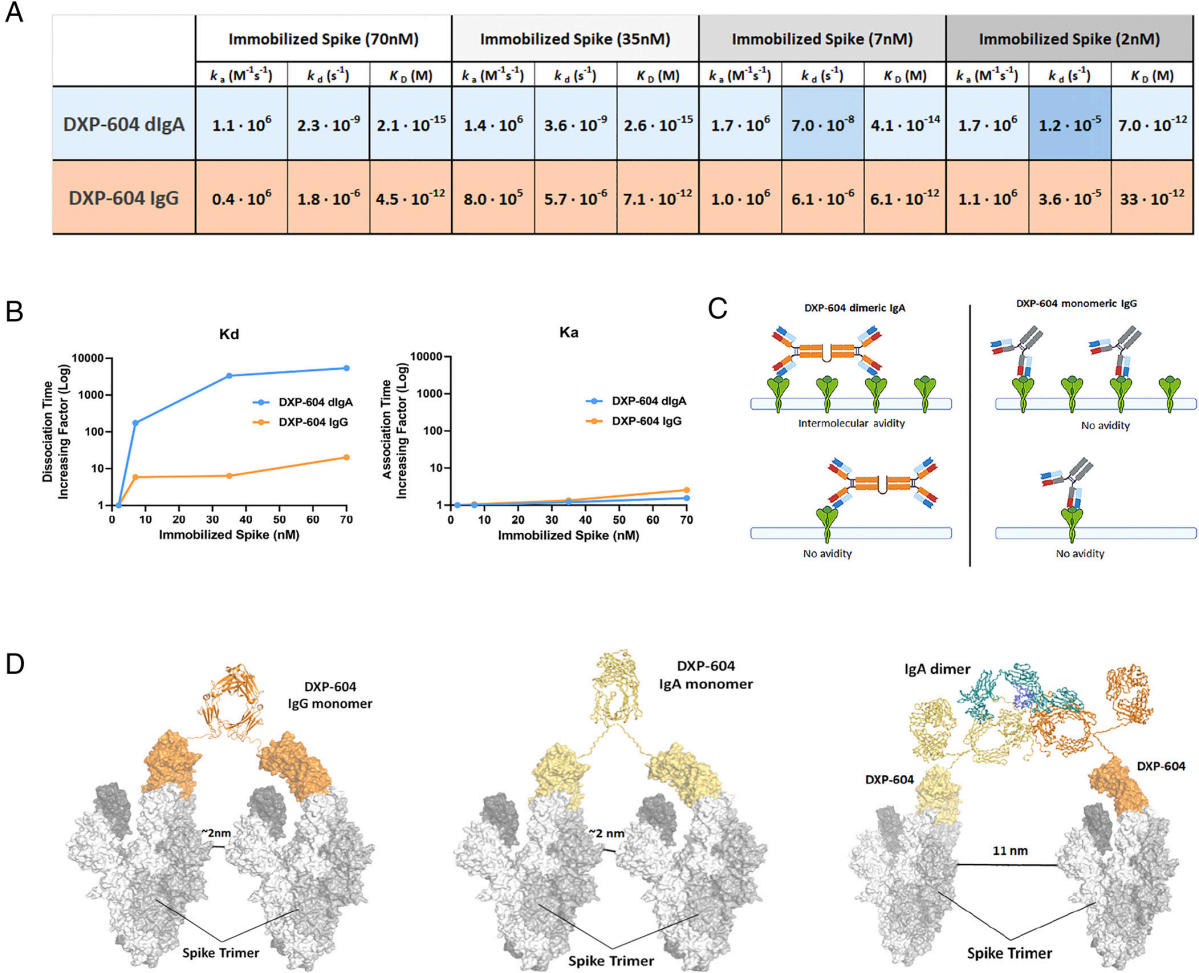
Increased neutralization potency of DXP-604 dimeric IgA is associated with increased avidity. (*A*) *k*_a_/*k*_d_ values obtained at different concentrations of immobilized antigen (Spike) for DXP-604 dimeric IgA1 and IgG forms. *k*_a_ remains equal across concentrations whereas *k*_d_ becomes ~1,000 times slower for the dIgA1, indicative of intermolecular avidity available only to the dIgA1. Shades of blue indicate the difference in *k*_d_ value for DXP-604 dIgA1. (*B*) Plots of *k*_d_ (*Left*) and *k*_a_ (*Right*) at different concentrations of immobilized spike, highlighting intermolecular avidity effects (slower dissociation, same association) for the dIgA1 (blue) in comparison to the IgG (orange). (*C*) DXP-604 dIgA1 and monomeric IgG have different binding modes that are available when high or low quantities of S-trimers are immobilized on the surface of the SPR chip. (*D*) Computational simulation showing inter-Spike linking by DXP-604 monomeric IgG and IgA1, and dimeric IgA1 antibodies. The predicted distance between S-trimers necessary for interlinking is indicated.

As the EC_50_ value obtained by ELISA does not measure affinity, and since DXP-604 dimeric and secretory IgA1 neutralized Omicron with a high potency, we further characterized the binding properties of this antibody. We used an avidity assay by surface plasmon resonance (SPR), to experimentally confirm that DXP-604 IgA, but not IgG, can simultaneously engage neighboring S antigens, resulting in intermolecular avidity effects and correspondingly slower dissociation rates (39). Structural analysis shows that only one DXP-604 Fab can bind to each S trimer due to light chain steric clashes, but it can prevent the binding of ACE2 to all three S monomers (*SI Appendix*, Fig. S2*C*). Therefore, no intramolecular avidity, involving two or more antibody arms on the same S trimer, is available for either IgA or IgG. Indeed, SPR shows equal association and dissociation rates for the two formats binding to S trimers immobilized at a low concentration on the SPR chip surface, approaching a “single molecule” distribution (Fig. 4 *A* and *B*). The situation changes when moving to higher concentrations of immobilized antigen, allowing intermolecular avidity due to higher density and correspondingly closer spatial distribution (Fig. 4*C*). At high antigen concentrations, the dissociation rate of IgA DXP-604 is ~1,000 times slower than that of the IgG, indicative of intermolecular avidity for the former but not the latter, due to intermolecular binding of the “distant” arms of the IgA, increased flexibility of the IgA hinge or both (Fig. 4 *A*–*C*). As a control, the association rates remain unchanged at increasing concentration of immobilized antigen, in agreement with the notion that avidity results in slower dissociation without affecting association rates (Fig. 4 *A* and *B*).

The S-trimers on the surface of SARS-CoV-2 float readily and are widely spaced with a mean a mean of 24 trimeric S protein per virus (40) at an average distance of 25 nm (41). Theoretically, IgG and mIgA1 antibodies may bind the RBD on two different S-trimers spaced by ~2 nm. Structural simulations show that dIgA1 and sIgA1 can bridge upon S-trimers ~11 nm apart (Fig. 4*D*). It is plausible that the dimeric forms are more likely to engage in intermolecular binding on the viral surface, especially if increased flexibility for the IgA is taken into account. Thus, the increased neutralizing potency of dIgA1 and sIgA1 appears to be, at least partly, due to increased avidity mediated by inter-S-trimer binding on the viral surface. However, other mechanisms such as intervirion aggregation, may also be involved (42).

### Dimeric IgA Is Protective Against Omicron BA.5 in a Mouse Model.

**Fig. 5.**
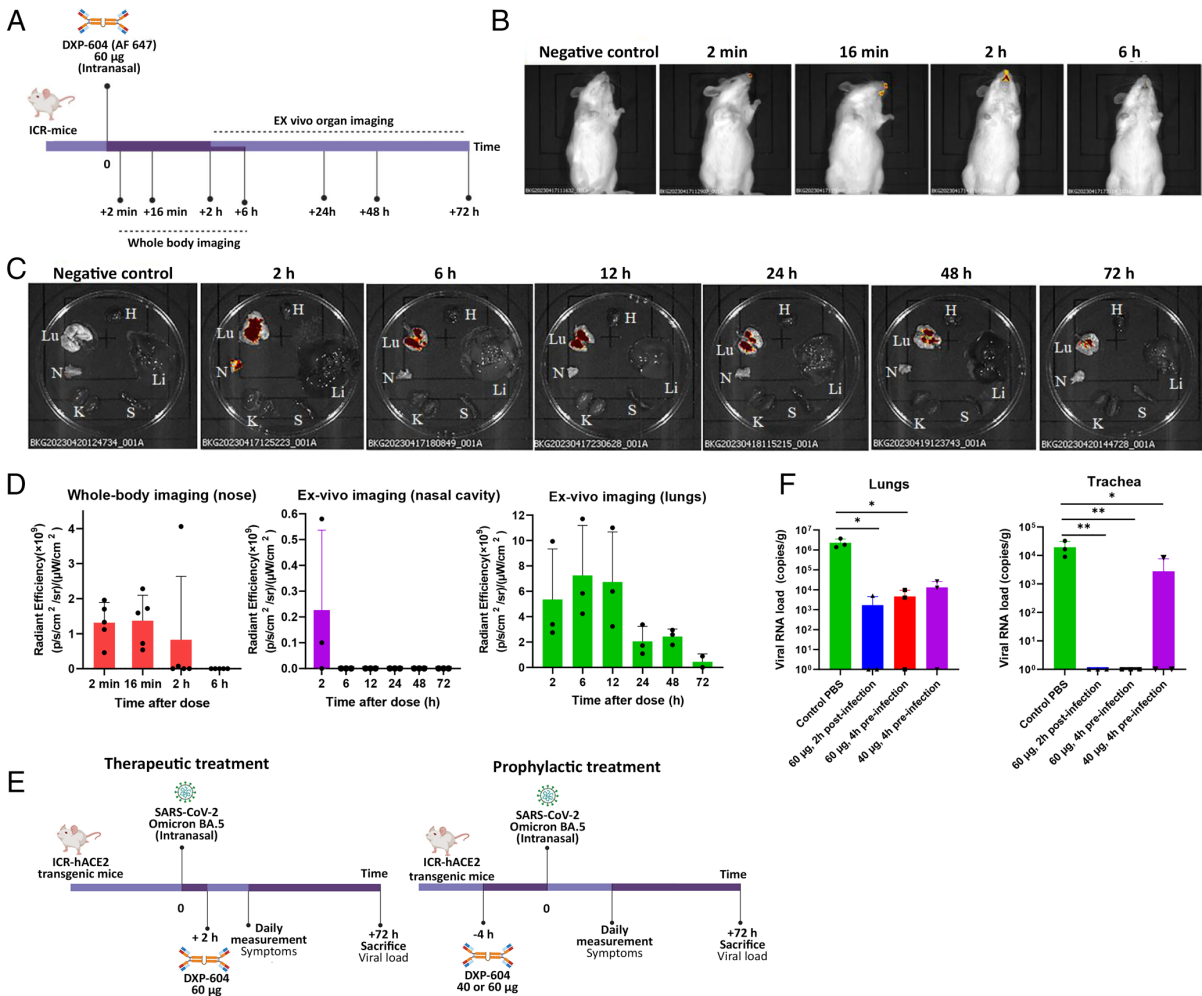
Intranasal administration of dimeric IgA in hACE2 mice is protective against Omicron BA.5. (*A*) Experimental design for the evaluation of antibody biodistribution after administration of 60 µg DXP-604 dIgA1 (labeled with Alexa Fluor 647). (*B*) Representative whole-body images. (*C*) Representative ex vivo images. N, nasal cavity; Lu, lungs and section of the trachea; H, heart; Li, liver; S, spleen; K, kidney. In (*B*) and (*C*), the negative control mice received PBS only. (*D*) Quantification of fluorescence signals. Data are presented as mean ± SD of five mice (whole-body imaging) or three mice (ex vivo nasal cavity and lung imaging). (*E*) Experimental design for the evaluation of DXP-604 dIgA1 using therapeutic and prophylactic intranasal administration. (*F*) Viral loads in the lung and tracheal tissues at 3 d post-infection of Omicron BA.5-infected mice after administration of a single dose of DXP-604 dIgA1 in a therapeutic (60 µg, 2 h post-infection) and prophylactic (40 or 60 µg, 4 h preinfection) setting. Viral loads are expressed as the mean ± SD for three mice. A two-sided unpaired *t* test was used. *P < 0.05 and **P < 0.01.

We first evaluated the biodistribution of DXP-604 dIgA1 (labeled with Alexa Fluor 647) following intranasal administration in Institute of Cancer Research (ICR) mice (Fig. 5*A*). After a single intranasal dose of DXP-604 dIgA1 (60 µg), the antibody remained detectable in the nasal cavity for a minimum of 2 h using whole-body imaging (Fig. 5*B*). Ex vivo organ imaging showed that DXP-604 dIgA1 was present in the nasal cavity in two out of three mice 2 h after administration and in the lungs of all dissected mice for at least 48 h (Fig. 5 *C* and *D*). No DXP-604 dIgA1 antibody was detected in other organs tested, including heart, liver, spleen, and kidney (Fig. 5*C*). These results indicate that intranasally administered DXP-604 dIgA1 mainly targets the respiratory tract, with persistence in the lungs for at least 48 h.

We subsequently evaluated the protective efficacy of DXP-604 dIgA1 through therapeutic and prophylactic intranasal administration in transgenic ICR mice expressing human ACE2 (hACE2) that were infected with Omicron BA.5 (50 µL, 10^5^ TCID50/mL) (Fig. 5*E*). The viral load in the lungs and tracheal tissues of untreated mice only given phosphate-buffered saline (PBS) was 10^6.37^ copies and 10^4.29^ copies/g, respectively, at 3 d postinfection (dpi). A single intranasal dose of DXP-604 dIgA1 (60 µg, ~2.2 mg/kg), 2 h after challenge with Omicron BA.5, significantly reduced the mean viral load to 10^3.22^ copies/g (a 3.15 log10-fold decrease) in lung tissues and to undetectable levels (a 4.29 log_10_-fold decrease) in the tracheal tissues of all three treated mice at 3 dpi (Fig. 5*F*). For prophylactic treatment, administration of 60 µg of DXP-604 dIgA1 (~2.9 mg/kg) 4 h before challenge with the virus significantly reduced the mean viral load to 10^3.66^ copies/g (a 2.71 log_10_-fold decrease) in lung tissues and to undetectable levels (4.29 log_10_-fold decrease) in tracheal tissues of all treated mice (*n* = 3) at 3 dpi (Fig. 5*F*). A lower prophylactic dose (40 µg, ~1.5 mg/kg) significantly reduced the viral load in the trachea only (a 0.84 log_10_-fold decrease). Together, our data show that prophylactic and therapeutic intranasal administration of DXP-604 dIgA1 is highly protective against Omicron infection in the respiratory tract in the hACE2 mouse model.

### Conversion to IgA1 and Dimerization Increases the Neutralization Potency Against Circulating Omicron Subvariants.

According to the World Health Organization (WHO), more than 300 sublineages of Omicron, mainly descendants from BA.2 and BA.5, are circulating globally (https://github.com/gerstung-lab/SARS-CoV-2-International), with XBB.1.5, XBB.1.9.1, XBB.1.9.2, XBB.1.16, and EG.5.1 currently being dominant in most parts of the world. Compared to BA.5, BQ.1 carries two additional mutations (K444T and N460K) in the RBD, while BQ.1.1 carries an additional RBD mutation (R346T). BA.2.75 carries three mutations (G446S, N460K, and a reversed (rev) mutation R493Q) in the RBD compared to BA.2, and BA.2.75.2 contains two additional mutations (R346T and F486S). The S protein of XBB has 14 mutations in addition to those found in BA.2, including 9 in the RBD (G339H, R346T, L368I, V445P, G446S, N460K, F486S, F490S, and rev R493Q), whereas sublineages have additional mutations (G252V for XBB.1; G252V and S486P for XBB.1.5, XBB.1.9.1 and XBB.1.9.2; G252V, S486P, and K478R for XBB.1.16; Q52H, G252V, F456L, and S486P for EG.5.1).

Since authentic viruses of the new subvariants were not yet available in our laboratories at the time of the study, we evaluated the neutralization activity of DXP-604 IgG and IgA1 against circulating subvariants, in addition to BA.1, BA.2, and BA.4/5, using pseudovirus assays. Dimeric and secretory IgA1 (IC_50_: < 0.0012 to 0.022 nM) improved the neutralization of BA.1, BA.2, and BA.4/5 Omicron subvariant pseudoviruses by 35.4- to 110-fold compared to monomeric IgG (IC_50_: 0.091 to 0.836 nM) (*SI Appendix*, Fig. S8). Furthermore, DXP-604 dIgA1 and sIgA1 increased the neutralizing activity up to 39.0-fold against BQ.1, BQ.1.1, and BA.2.75 (IC_50_: 0.026 to 9.75 nM) compared to IgG (*SI Appendix*, Fig. S8). However, DXP-604 IgA1 forms could not restore neutralization activity against BA.2.75.2 and XBB.1. DXP-604 IgG and IgA forms did not bind the RBD protein of XBB.1.5 and XBB.1.16 in ELISA and were thus not tested for neutralization.

Interestingly, 01A05 IgG, which showed no binding to BA.2 in ELISA (*SI Appendix*, Fig. S5*A*), neutralized BA.2.75.2 (IC_50_: 12.08 nM) and the effect was greatly improved by 180- to 1,012-fold using mIgA1, dIgA1, or sIgA1 (*SI Appendix*, Fig. S9). Furthermore, an increase in neutralization activity (by >95-fold) against XBB.1, XBB.1.5, and XBB.1.16 was observed for 01A05 mIgA1, dIgA1, or sIgA1 (IC50: 0.06 to 0.35 nM) compared to IgG (IC_50:_ 7.5 to >66.7 nM), which poorly neutralizes these variants. As previously observed with a few other monoclonal antibodies (43, 44), the R493Q reversion mutation in BA.2.75.2, XBB.1, XBB.1.5, and XBB.1.16 at least partially restored the 01A05 neutralizing epitope found in the ancestral SARS-CoV-2 (*SI Appendix*, Fig. S3*A*).

Since many Omicron subvariants with multiple convergent mutations are simultaneously circulating in different parts of the world (10) and new subvariants such as BA.2.86 are emerging, our results suggest that a cocktail of dimeric or secretory IgA1 antibodies, including DXP-604 or 01A05 described here and those newly identified broad neutralizers (10, 45, 46), would be necessary to neutralize most, if not all, emerging Omicron subvariants and future VOCs.

## Discussion

SARS-CoV-2 primarily infects the upper respiratory tract, where the mucosal immune response is expected to be mainly induced in the nasopharynx, via the tonsils and adenoids, collectively referred to as the nasopharynx-associated lymphoid tissue. Systemic immunization has generally been considered ineffective in generating protective mucosal immune responses, although certain antigen and adjuvant combinations can elicit mucosal immune responses, including sIgA antibodies (47). The mechanism of this induction remains poorly understood and may involve the migration of vaccine antigens, antigen-loaded antigen-presenting cells, and/or antigen-specific B cells to mucosal-associated lymphoid tissues (47, 48). In this study, the inactivated and/or mRNA vaccines induced salivary IgA anti-RBD antibodies in 60% of individuals but at a low level in comparison to individuals with BTI, particularly against the Omicron variants. Furthermore, additional vaccine doses did not boost the salivary RBD-specific IgA response. Thus, while vaccines administered through peripheral injection can generate low/modest levels of IgG and monomeric IgA antibodies at the mucosal surfaces of the upper respiratory tract, they are not particularly effective at inducing secretory IgA antibodies (49). Furthermore, the recruitment of circulating SARS-CoV-2 specific memory B cells and T cells can reduce COVID-19 symptoms. However, it is not particularly effective in preventing transmission of SARS-CoV-2. We have previously shown that a high level of mucosal RBD-specific IgA antibodies induced by vaccination might be associated with protection against BTI (28). In the present study, we have further shown that BTI gives rise to a higher IgA response than mRNA or inactivated vaccines. Therefore, we might expect a higher degree of protection against new infections in individuals with BTI compared to those given only vaccines. This is consistent with several recent studies that have shown that the protection against Omicron BTI is largely mediated by mucosal IgA (22, 50). The distinct “immunization” routes following natural infection (airway mucosal route) and intramuscular vaccination (systemic route) may explain the higher immune response, including the sIgA response, and greater protection associated with infection-acquired immunity compared to vaccine-acquired immunity (51). These findings support the importance of the mucosal immune system in defending against SARS-CoV-2, especially the latest Omicron variants. They also emphasize the need for the development of vaccines that can elicit a stronger mucosal IgA response (16, 22, 52) and therapies based on intranasally administered antibodies. The development of IgA prophylaxis, specifically designed to target the respiratory tract, is expected to be more efficient at limiting virus transmission compared to systemic immunization.

Omicron lineages and subvariants are currently circulating worldwide, and they carry mutations that confer resistance to most potent neutralizing antibodies, including those clinically approved (10, 53). In agreement with previous studies, we show that only DXP-604, one of our identified SARS-CoV-2 RBD-neutralizing antibodies encoded by IGHV3-53, can broadly neutralize most SARS-CoV-2 variants and selected subvariants (10). This outcome is probably achieved through high affinity and ACE2-mimicking interactions via light chain complementarity-determining regions, especially hydrogen bonds formed with RBD G502 and Q498. Although the neutralizing activity of DXP-604 IgG against Omicron BA.1, BA.2, and BA.5 (2, 53) was lower than that of LY-CoV1404 (bebtelovimab) (53, 54) and other IgG antibodies in preclinical or clinical development (55, 56), DXP-604 dIgA1 and sIgA1 showed a 24- to 72-fold increase in potency against the BA.1, BA.2, and BA.5 variants (IC_50_: 0.05 to 0.87 nM), reaching the level of one of the most potent antibodies, LY-CoV1404 (IC_50_: 0.08 to 0.1 nM) (*SI Appendix*, Table S2). Contrary to LY-CoV1404 and other antibodies approved for clinical use (4, 10), DXP-604 dIgA1 and sIgA1 could also neutralize BQ.1 and BQ.1.1. However, DXP-604 was recently shown not to be able to neutralize sublineages carrying the mutation in position 486 of the S protein, explaining its inability to neutralize BA.2.75.2 and XBB sublineages (10). Nevertheless, 01A05 dIgA1 and sIgA1 strongly improved neutralization potency against BA.2.75.2, XBB.1, XBB.1.5, and XBB.1.16 and most likely also against XBB.1.9.1 and XBB.1.9.2 which share the same S protein amino acid sequence as XBB.1.5, suggesting that a combination of dimeric or secretory IgA1 antibodies could be used to neutralize a wider range of variants with a higher potency. Additional IgG antibodies could also be further engineered into IgA formats, for example, the recently described SA55, that broadly neutralizes all the variants tested thus far, including the recently emerging variants EG.5 and BA.2.86 (45, 57).

The subclass proportions vary with mucosal site but typically range from 80 to 90% IgA1 in nasal and male genital secretions, 60% IgA1 in saliva, to 60% IgA2 in colonic and female genital secretions (19). IgA1 and IgA2 differ mostly in the hinge region, which is significantly longer (13 aa) in IgA1. As a result, IgA1 is more like a T-shaped molecule (58), whereas IgA2 resembles the more rigid classical Y-shape of IgG (59), as revealed by X-ray crystal structures. Consistent with previous studies, a switch to monomeric IgA may result in an increase in the neutralizing capacity of certain antibodies, such as DXP-604, and against some variants (60) compared to that of IgG. The longer IgA1 hinge may confer increased flexibility and allow two Fabs to reach two RBDs in the trimer at the same time, thus enhancing SARS-CoV-2 neutralization in vitro compared to that by their IgG counterparts. However, computational modeling showed that although 01A05 and XG014 Fabs each simultaneously can bind two RBDs on the same trimeric S protein, DXP-604 Fabs cannot bind more than one site due to steric hindrance caused by the light chain (*SI Appendix*, Fig. S2). Alternatively, the increased flexibility in the hinge region could help the binding of one Fab fragment to RBD or promote linking of S on two different virus particles. We tested DXP-604 antibodies in the IgA2 format and found that DXP-604 mIgA1 neutralizes Omicron BA.2 and BA.5 with more than 15-fold higher potency than DXP-604 mIgA2. However, considering the higher proportion of IgA2 in the intestinal tract and its increased resistance to bacterial proteases due to its shorter hinge, dimeric, or secretory IgA2 might still be useful for oral delivery to prevent fecal–oral transmission or reduce gastrointestinal symptoms (61).

The transgenic hACE2 mouse model has been used previously in research to study SARS-CoV-2 infection and evaluate potential vaccines and therapeutic agents (32, 45). Intranasal delivery of IgM (3.5 mg/kg) (38) and IgG (3 mg/kg) (62) antibodies after infection with SARS-CoV-2 (Beta or Omicron BA.2) was previously shown to reduce the viral load in the lungs of hACE2 expressing mice. We showed here, for the first time, that a therapeutic or prophylactic intranasal administration of dIgA1, confers significant protection against Omicron BA.5 in the hACE2 transgenic mouse model and considerably reduces the viral load in both the lung and trachea. The higher conferred protection in the trachea compared to the lung could be due to the biodistribution of the antibodies following intranasal administration. However, as previously observed for other antibody isotypes, dimeric IgA showed the longest retention in the lungs according to ex vivo imaging (38, 63). While the humanized ACE2 mouse model can provide valuable data on evaluating the neutralization activity of antibodies in vivo, it is essential to further test these antibodies in other animal models to determine their efficacy in reducing SARS-CoV-2-induced lung damage or inflammation. Subsequently, human clinical trials are necessary to confirm their safety, biodistribution, and efficacy when administered via nasal spray or drops.

The half-life of sIgA on the mucosal surface of human is generally believed to be relatively short, ranging from a few hours to a couple of days (64, 65). Substances applied to the nasal mucosa are largely removed by mucociliary clearance activity (66). However, it has been shown that following intranasal delivery of IgA (about 0.5 mg/kg) in macaques, once daily for 2 d before and 4 d after RSV challenge, IgA could be demonstrated in the nasal cavity for at least 24 h after the last application. This approach was found to nearly completely abrogate the infection in the upper and lower respiratory tract (67). Self-administration of human IgA antibodies via nasal spray twice daily for 17 d also significantly reduced the incidence of upper respiratory tract infections in elite skiers (68).

Thus, based on the published studies, we expect the IgA antibodies to offer protection for at least 24 h in humans. In fact, sIgA could reside for a longer period on the mucosal surface, as the SC can protect IgA from proteases (69). Furthermore, a prolonged presence of IgA in the nasal cavity could also be achieved by the use of delivery systems, such as liposomes or nanoparticles, or by increasing the viscosity of the formulation using various polymers (70, 71). Thus, additional strategies, such as modifying the antibody concentration or optimizing the formulation for extended duration of antibodies on the nasal mucosa, may be used to improve the protection conferred by nasal sprays in the future. The persistence of the DXP-604 antibodies should thus be tested in a phase I trial using GMP preparations. Once developed, nasal spray or drops have the potential for self-administration on a daily basis, even twice daily, either as a preventive measure against transmission, or shortly before engaging in activities involving public spaces during COVID-19 waves. Medical staff in direct contact with COVID-19 patients could also utilize nasal spray or drops for added protection against transmission of the virus. Additionally, they can serve as a treatment option for individuals who have tested positive for COVID-19, effectively preventing the virus from establishing an infection in the lungs. Individuals at high risk of developing severe illnesses or experiencing prolonged viral shedding in the lungs or intestines, such as the elderly and immunocompromised patients, particularly those who are HIV-positive, could also benefit from such antibody therapy (72). When administered either intranasally or orally, this approach could help reduce the spread of the virus and its potential evolution.

Thus, intranasal delivery of dimeric or secretory DXP-604 IgA in combination with other IgA antibodies directly to the site of infection may be an effective approach to achieve immediate protection against SARS-CoV-2 infection, which is needed at a small window for intervention, such as prevention in high-risk settings or postexposure prophylaxis. IgA does not activate complement and may inhibit complement activation induced by IgG and IgM, thus reducing inflammation (15).

A formulation of dIgA or sIgA anti-SARS-CoV-2 antibodies may eventually be applied by susceptible individuals themselves with or without medical supervision and thus has a wider coverage, which is especially important in resource-poor areas. Nasal delivery of antibodies is likely to be carried out using low doses, since the drug is administered locally, where it is most needed, in contrast to the larger amounts needed for systemic application. To ensure sustainable production of the therapeutic agent, these antibodies can be expressed in plants (rice, tobacco) (73). Produced in this way, recombinant sIgA-based passive immunotherapies or prophylactics could represent extremely effective tools for the control of SARS-CoV-2 infections.

## Material and Methods

### Study Design.

The objectives of the study were to evaluate the mucosal immune response to SARS-CoV-2 in vaccinated individuals as compared to those with mild BTI and to develop dimeric and secretory IgA1 antibodies for mucosal prophylactic and therapeutic treatments. We tested the presence of antibodies against the RBDs of G614 (wild-type) and Omicron lineages BA.1, BA.2, and BA.4/5 in saliva and plasma of individuals with different types of vaccinations. The anti-RBD antibody levels and total IgA levels were measured by ELISA.

We subsequently engineered and characterized recombinant monomeric, dimeric, and secretory IgA1 antibodies derived from four neutralizing IgG mAbs (01A05, rmAb23, DXP-604, and XG014) targeting the RBD domain of the spike protein. The anti-RBD IgG and IgA1 antibody forms were produced and tested for binding to RBD in ELISA and for neutralizing activity using authentic viruses and pseudovirus. Computational modeling for predicting the antibody structure and antibody-RBD interaction was performed. We used an avidity assay by SPR to experimentally confirm that the dimeric IgA antibody can simultaneously engage two RBDs on different S proteins.

We assessed the biodistribution of DXP-604 dIgA1, labeled with Alexa Fluor 647, following intranasal administration in Institute of Cancer Research (ICR) mice (8 to 10 wk old). We also evaluated the protective efficacy of DXP-604 dIgA1 in ICR-hACE2 mice (8 to 10 wk old) against challenge with Omicron BA.5 (SARS-CoV-2/human/CHN/GD-5/2022, GenBank: OP678016) using intranasal therapeutic and prophylactic administration. Detailed descriptions of the experimental procedures are provided in *SI Appendix*.

### Ethical Considerations.

The study for evaluating the immune response and isolation of monoclonal antibodies in participants was approved by the ethics committee of the institutional review board of Stockholm. A written, informed consent was obtained from each participant.

The procedures to evaluate the biodistribution of IgA following intranasal administration were approved by Institutional Animal Care and Use Committee, Kunming Institute of Zoology, Chinese Academy of Sciences.

The animal study to evaluate efficacy of DXP-604 dIgA1 was performed in an animal biosafety level 3 (ABSL3) facility using HEPA-filtered isolators. Specific-pathogen-free (SPF) ICR-hACE2 mice, 8 to 10 wk old (18 to 32 g), were provided by the Institute of Medical Experimental Animals, Chinese Academy of Medical Sciences. The procedures were approved by the Institute of Laboratory Animal Sciences (ILAS), Chinese Academy of Medical Sciences (CAMS), and Peking Union Medical College (PUMC).

## Supplementary Material

Appendix 01 (PDF)

Dataset S01 (XLSX)

## Data Availability

All study data are included in the article and/or supporting information. All other data and material are available from the corresponding authors upon reasonable request.
