# Supplementary material for: Conversion of monoclonal IgG to dimeric and secretory IgA restores neutralizing ability and prevents infection of Omicron lineages
Source: Proc Natl Acad Sci U S A. 2024 Jan 9;121(3):e2315354120. doi: 10.1073/pnas.2315354120 (PMC10801922; doi:10.1073/pnas.2315354120)
Supplement: Supplementary file 1 — Appendix 01 (PDF) [file pnas.2315354120.sapp.pdf]

PNAS

Supporting Information for

Conversion of monoclonal IgG to dimeric and secretory IgA restores neutralizing ability and prevents infection of Omicron lineages

Harold Marcotte, Yunlong Cao, Fanglei Zuo, Luca Simonelli, Josè Camilla Sammartino, Mattia Pedotti, Rui Sun, Irene Cassaniti, Marie Hagbom, Antonio Piralla, Jinxuan Yang, Likun Du, Elena Percivalle, Federico Bertoglio, Maren Schubert, Hassan Abolhassani, Natalia Sherina, Concetta Guerra, Stephan Borte, Nima Rezaei, Makiko Kumagai-Braesch, Yintong Xue, Chen Su, Qihong Yan, Ping He, Caroline Grönwall, Lars Klareskog, Luigi Calzolai, Andrea Cavalli, Qiao Wang, Davide F. Robbiani, Michael Hust, Zhengli Shi, Liqiang Feng, Lennart Svensson, Ling Chen, Linlin Bao, Fausto Baldanti, Junyu Xiao, Chuan Qin, Lennart Hammarström, Xing Lou Yang, Luca Varani, Xiaoliang Sunney Xie, Qiang Pan-Hammarström

Xiaoliang Sunney Xie and Qiang-Pan Hammarström
Email: sunneyxie@biopic.pku.edu.cn, qiang.pan-hammarstrom@ki.se

This PDF file includes:

Supporting text
Figures S1 to S9
Tables S1 and S2
References

Other supporting materials for this manuscript include the following:

Datasets S1

Supporting Information Text

Supporting methods

Study subjects.

Study inclusion criteria included subjects who were older than 18 years of age, received inactivated and/or mRNA vaccines with a documented vaccination history (type of vaccine, number of doses, interval between the doses, days after the latest dose, and infection history), and were willing and able to provide written informed consent. The study included 133 paired saliva and plasma samples from 89 healthy volunteers (63% females, median age of 32 years) in Sweden in 2021-2022 who received two or three doses of inactivated vaccine (CoronaVac, Sinovac or BBIBP-CorV, Sinopharm), 1 to 3 doses of an mRNA vaccine (BNT162b2, Pfizer–BioNTech or mRNA-1273, Moderna) or a combination of both (two doses inactivated vaccine followed by a heterologous mRNA boost), some of whom had experienced BTIs during the Omicron BA.1, BA.2 and BA.5 waves (from Dec 2021 to June 2022). A group receiving one or two doses of mRNA vaccine after SARS-CoV-2 infection (during the G614 wave) was also included (*SI Appendix,* Table S1). Samples were collected 5-198 days (median day 20.5) after each mRNA dose including after mRNA heterologous boost, 6-92 days (median day 51) after doses 2 and 3 of inactivated vaccine and 8-43 days (median day 19) after BTI (*SI Appendix,* Table S1). Infection was confirmed when an individual tested positive for antigen or qPCR test. All cases of infection were mild, and none required outpatient visits or hospitalization due to clinical complications. Data from a subset of the samples from donors receiving the mRNA vaccine (2nd and 3rd doses) and BTI after the mRNA vaccine were described previously (1) and included for comparison. Saliva and plasma samples from prevaccinated, uninfected healthy donors in our cohort were also included as negative controls (n = 7) (1). To confirm that salivary IgA was a reliable marker of mucosal immunity, nasal swab samples were also taken from individuals who were either vaccinated against SARS-CoV-2 (n = 2

with either 3 doses mRNA of vaccines or two dose of inactivated vaccine plus 1 dose mRNA vaccine) or had BTI (*n* = 13) for comparison of IgA levels. These samples were collected 18 to 19 months after their last vaccine dose or 3 to 20 months after experiencing BTI. The study for evaluating the immune response and isolation of monoclonal antibodies in participants was approved by the ethics committee of the institutional review board of Stockholm (Dnr 2022-00676-01 and 2020-01612). A written, informed consent was obtained from each participant.

Detection of antibodies specific to SARS-CoV-2 RBD.

To assess anti-RBD binding activity, high-binding Corning half-area plates (Corning #3690) were coated with RBD derived from G614, BA.1, BA.2 or BA.4/5 (1.7 μg/ml) in PBS and incubated overnight at 4°C. Dilutions of saliva or nasal fluid in PBS with 5% skim milk supplemented with 0.1% Tween 20 were added, and the plates were subsequently incubated for 1.5 hours at room temperature. The plates were then washed and incubated for 1 hour with horseradish peroxidase (HRP)-conjugated goat anti-human IgM (Invitrogen #A18835), goat anti-human IgA (Jackson #109-036-011), or goat anti-human IgG (Invitrogen #A18805) antibodies (all diluted 1:5000 in PBS supplemented with 5% skim milk and 0.1% Tween 20). For detection of secretory immunoglobulins (sIgA and sIgM), plates were incubated for 1 hour with HRP-conjugated goat anti-secretory component antibodies (Nordic–MUbio, #GAHu/SC/PO) diluted 1:1000 in PBS supplemented with 5% skim milk and 0.1% Tween 20. The bound antibodies were visualized using tetramethylbenzidine substrate (Sigma #T0440). The colour reaction was stopped with 0.5 M H_2SO_4 after 10 min of incubation, and the absorbance was measured at 450 nm in an ELISA plate reader (Tecan). Each sample was tested in duplicate, and the mean OD_{450} nm values were used to calculate the concentration of specific and total antibodies, with the exception of a few samples that could not be run in

duplicate due to insufficient amount of saliva. Plasma anti-RBD IgA and IgG levels were measured as previously described (2).

Plasma IgA, and nasal and salivary antibody levels are reported as arbitrary units (AU)/ml based on a standard curve generated with data derived from a serially diluted highly positive in-house serum pool. Plasma and salivary IgG levels were expressed as binding antibody units (BAU)/ml after calibrating in-house standards to the WHO International Standard for anti-SARS-CoV-2 Ig (NIBSC, 20/136) (3, 4). For sIg, serial dilutions of human monoclonal secretory IgA anti-RBD antibodies were used for the generation of a standard curve and measurement of concentrations (ng/ml). Salivary IgA anti-RBD antibodies were normalized according to the total level of salivary IgA (AU/µg total IgA) to compensate for the different salivary flow rates between individuals. The positive cut-off was calculated to be 2 standard deviations (2SD) higher than the mean of a pool of samples taken from prevaccinated and noninfected individuals.

Detection of total IgA.

To assess total IgA, high-binding Corning half-area plates (Corning #3690) were coated overnight at 4°C with polyclonal goat anti-human IgA (Southern Biotech, #C5213-R466) (2 µg/ml) in PBS. Dilutions of saliva in PBS supplemented with 5% skim milk and 0.1% Tween 20 were added, and the plates were subsequently incubated for 1.5 hours at room temperature. The plates were then washed and incubated with HRP-conjugated polyclonal goat anti-human IgA (Jackson #109-036-011) at a dilution of 1:15000. The bound antibodies were detected as described above. Serial dilutions of human monoclonal IgA were used for the generation of standard curves and measurement of concentrations (ng/ml).

Production of SARS-CoV-2 RBD protein.

The RBDs of G614, Alpha, Beta, and Omicron (BA.1, BA.2, BA.4/5) variants were ordered as GeneString from GeneArt (Thermo Fisher Scientific). All sequences of the RBD (aa 319-541 in GenBank: MN908947) were inserted into a NcoI/NotI compatible variant of an OpiE2 expression vector carrying the N-terminal signal peptide of the mouse Ig heavy chain and a C-terminal 6×His-tag. RBD of G614, Beta, Delta and Omicron were expressed in a baculovirus-free expression system in High Five insect cells and purified on HisTrap Excel columns (Cytiva) followed by size-exclusion chromatography on 16/600 Superdex 200-pg columns (Cytiva) (5).

Isolation of IgG antibodies by sorting RBD-binding memory B cells.

The 01A05 IgG monoclonal antibody was generated from single-sorted RBD-binding B cells isolated from the blood of convalescent patients infected with the G614 strain. B cells were isolated from peripheral blood mononuclear cells (PBMCs) using the EasySep™ Human B Cell Isolation Kit (StemCell Technology), resuspended in 500 µl PBS, 1% human serum (PBS-HS), and labelled for flow cytometry. First, cells were incubated with RBD protein (labelled with either anti-His tag conjugated to allophycocyanin (APC) or phycoerythrin (PE)) for 60 min at 4°C, washed in PBS-HS, centrifuged (8 min, 400g, 4°C), resuspended in 500 µl PBS-HS. Next, cells were stained with 7-aminoactinomycin D. Viability Staining Solution (BioLegend) to detect dead/dying cells, and anti-CD3 (FITC), anti-CD14 (FITC), and anti-CD19 (BV421). Single viable $CD3^-$/$CD14^-$/$CD19^+$ RBD-binding cells were sorted into 96-well PCR plates containing 5 µl 0.5×PBS, 10 mM DTT, 8 U RNAsin Ribonuclease Inhibitors /well (Promega, Madison, WI, USA), and stored at -80°C until further processed. Variable region Ig cDNA was synthesized by reverse transcription and amplified using multiplex PCR, as previously described (6). Paired Ig heavy (IgH) and light (IgL) chains were sequenced

(Eurofins Genomics) and annotated using IgBLAST, and IMGT/V-QUEST. Selected sequences were cloned and expressed as IgG1 in Expi293 cells.

XG014 (7, 8) and DXP-604 (9–11) IgG were previously isolated by sorting RBD-binding memory B cells from convalescent patients infected with the Wuhan strain. rmAb23 was previously isolated using an antibody repertoire prepared by sequencing PBMCs from patients infected with the Wuhan strain followed by matching of the VH3-53-J6 heavy chain, highly shared among COVID-19 patients, with a common IGKV1-9 light chain to produce recombinant antibodies (12).

Cloning of neutralizing IgG antibodies to generate the IgA forms.

The heavy and light chain variable genes of DXP-604, XG014, 01A05 and rmAb23 neutralizing IgG antibodies were cloned into separate pcDNA 3.4 vectors to mediate fusion to an IgA1 constant region and a light chain constant region gene (kappa for 01A05, rmAb23 and DXP-604 and lambda for XG014), respectively (GenScript). The J-chain and SC genes were cloned into separate pcDNA 3.4 expression plasmids for the assembly of dimeric and secretory IgA1.

Production and purification of antibodies.

The IgG and IgA1 antibodies 01A05, rmAb23, XG014 and DXP-604 were produced by transfection of HD CHO-S (Chinese Hamster Ovary) cells with plasmids in a 30-ml volume (GenScript). Monoclonal IgA1 antibodies were produced in CHO cells transiently transfected with two plasmids expressing a heavy and light chain. For the expression of dimeric and secretory IgA1 antibodies, cells were cotransfected with plasmids carrying the J-chain and SC. The IgG and IgA1 antibodies were purified by single-step affinity chromatography using

immobilized protein A (MabSelect SuRe™ LX, Cytiva) or anti-IgA antibody (CaptureSelect™ IgA Affinity Matrix), respectively (GenScript).

Binding of monoclonal and recombinant antibodies specific to SARS-CoV-2 RBD.

To assess the anti-RBD IgG or IgA binding activity, high-binding Corning half-area plates (Corning #3690) were coated with RBD derived from G614, Beta, Delta and Omicron (BA.1, BA.2, BA.4/5) (1.7 μg/ml) in PBS and incubated overnight at 4°C (3). Serial dilutions of antibody in PBS with 0.1% bovine serum albumin (BSA) were added, and the plates were subsequently incubated for 1.5 hours at room temperature. The plates were then washed and incubated with HRP-conjugated goat anti-human IgG (Invitrogen #A18805) or goat anti-human IgA (Jackson #109-036-011) (diluted 1:15000 in 0.1% BSA-PBS) followed by tetramethylbenzidine substrate. For each sample, the EC_{50} values were calculated using four-parameter nonlinear regression GraphPad Prism 7.04 software (2).

Pseudovirus neutralization assay based on the VSV platform.

The genes encoding the full S protein of G614, Alpha, Beta, Gamma, Delta, Kappa, Delta plus, Mu, Lambda, Iota, Omicron (BA.1, BA.2, BA.3, BA.1/BA.2 subvariants BA.1.1 (BA.1+R346K), BA.2.12.1 (BA.1+L452Q+S704L) and BA.2.13 (BA.1+L452M)) and clade 1b SARS-CoV-2 related sarbecoviruses (RaTG13 and Pangolin-GD) were cloned into the pcDNA3.1 vector. S pseudotyped virus was prepared based on a VSV pseudotyped virus production system (13). After transfection and culturing, the supernatant containing pseudotyped virus was harvested, filtered, and diluted to obtain the same particle number across samples, as determined based on quantitative analysis by RT–PCR, and frozen at −80°C for further use. Pseudovirus neutralization assays were performed using the Huh-7 cell line (Japanese Collection of Research Bioresources [JCRB], 0403) or human embryonic

kidney 293T cells overexpressing human ACE2, also called 293T-hACE2 cells (Sino Biological Company). Monoclonal antibodies were serially diluted in Dulbecco's modified Eagle's medium (DMEM) (HyClone, SH30243.01) and mixed with pseudovirus in 96-well plates. After the mixture was incubated for 1 hour in a 37°C incubator with 5% CO_2, trypsinised Huh-7 cells or 293T-hACE2 cells were seeded. After incubation, the supernatant was discarded, D-luciferin reagent (PerkinElmer, 6066769) was added to avoid a light reaction, and the luminescence value was detected with a microplate spectrophotometer (PerkinElmer, Ensight, 6005290). Each experiment was performed in triplicate, and the mean neutralization (%) values are presented in the figure. The IC_{50} was determined by a four-parameter logistic regression model.

Pseudovirus neutralization assay based on the HIV platform.

The human codon-optimized gene coding for the S protein of G614, BA.1, BA.2 and BA.4/5 lacking the C-terminal 19 codons ($S_{\Delta19}$) was synthesized by GenScript. The $S_{\Delta19}$ gene of BA.2.75, BA.2.75.2, BQ.1, BQ.1.1, XBB.1, XBB.1.5, and XBB.1.16 was constructed by site-directed mutagenesis (QuikChange Multi Site-Directed Mutagenesis Kit, Agilent) using the BA.2 or BA.4/5 $S_{\Delta19}$ gene as a template. To generate (HIV-1/NanoLuc2AEGFP)-SARS-CoV-2 particles, three plasmids were used, with a reporter vector (pCCNanoLuc2AEGFP), HIV-1 structural/regulatory proteins (pHIV$_{NL}$GagPol) and SARS-CoV-2 $S_{\Delta19}$ carried by separate plasmids as previously described (14). 293FT cells were transfected with 7 μg of pHIV$_{NL}$GagPol, 7 μg of pCCNanoLuc2AEGFP, and 2.5 μg of pSARS-CoV-2-$S_{\Delta19}$ carrying the $S_{\Delta19}$ gene from G614 or Omicron variants (at a molar plasmid ratio of 1:1:0.45) using 66 μl of 1 mg/ml polyethylenimine (PEI).

Tenfold serially diluted monoclonal antibodies were incubated with pseudovirus carrying the S protein from SARS-CoV-2 (G614, BA.1, BA.2, BA.4/5, BA.2.75, BA.2.75.2, BQ.1, BQ.1.1, XBB.1, XBB.1.5, and XBB.1.16) for 1 hour at 37 °C. The mixture was subsequently incubated with 293T-hACE2 cells for analyses of G614 or Omicron pseudoviruses for 48 hours, after which the cells were washed with PBS and lysed with Luciferase Cell Culture Lysis reagent (Promega). NanoLuc luciferase activity in the lysates was measured using the Nano-Glo Luciferase Assay System (Promega) with a Tecan Infinite microplate reader. The relative luminescence units were normalized to those derived from cells infected with the pseudotyped virus in the absence of monoclonal antibodies. Each experiment was performed in duplicate, and the mean neutralization (%) values are presented in the figures. The IC_{50} values for the monoclonal antibodies were determined using four-parameter nonlinear regression (the least squares regression method without weighting) (GraphPad Prism 7.04 software).

Microneutralization assay.

The SARS-CoV-2 G614 strain and VOCs (Alpha, Beta, Delta, and Omicron BA.1, BA.2 and BA.5) were isolated from patients in Pavia, Italy, and identified by next-generation sequencing. The neutralizing activities of the antibodies were determined via microneutralization assays (15). Briefly, 50 µl of an antibody, starting at 25 µg/ml and increased in a twofold dilution series, was mixed in a flat-bottom tissue culture 96-well microtiter plate (COSTAR, Corning Incorporated) with an equal volume containing a 100 median tissue culture infectious dose (TCID50) of a SARS-CoV-2 strain that had been previously titrated. All dilutions were performed using Eagle's minimum essential medium to which 1% (w/v) penicillin, streptomycin and glutamine and 5 µg/ml trypsin had been added. After 1 hour of incubation at 33°C in 5% CO_2, VERO E6 cells (VERO C1008 [Vero 76, clone

E6, Vero E6]; ATCC® CRL-1586™) were added to each well. After 3 days of incubation, the cells were stained with Gram's crystal violet solution (Merck) plus 5% formaldehyde (40% m/v) (Carlo Erba S.p.A.) for 30 min. Microtiter plates were then washed in tap water and analyzed to evaluate the degree of cytopathic effect compared to untreated controls. Each experiment was performed in triplicate, and the mean neutralization (%) values are presented in the figures. The IC_{50} was determined using four-parameter nonlinear regression (GraphPad Prism).

Detection of virus by immunofluorescence following neutralizing antibody assay.

DXP-604 monomeric IgG and monomeric, dimeric, and secretory IgA1 antibodies, each at a concentration of 3.3 nM, were mixed with an equal volume of Omicron BA.1 (200 plaque-forming units (PFU) per 100 µL) in DMEM supplemented with 2% fetal calf serum (FCS) and gentamycin and incubated at 37°C for 1 hour. Next, confluent VeroE6 cells in 96-well plates were washed twice with serum-free DMEM, and the cells were infected with 100 µl of a mAb-virus mix or with virus and no antibodies for 1 hour at 37°C with 5% CO_2. The cells were washed twice, and 100 µL of DMEM with 2% FCS and gentamycin was added to each well. After 9 hours of infection, the cells were fixed with 4% formaldehyde overnight. The cells were washed once with PBS and permeabilized with 0.2% Triton-X in PBS for 15 min at room temperature. Nonspecific binding was blocked with 3% BSA in PBS at 37°C for 1 hour. A primary antibody (mouse anti-dsRNA) at a 1:200 dilution with PBS containing 1% BSA was added and incubated for 2 hours at 37°C. After 3 washes, secondary goat anti-mouse Alexa 488 (Jackson ImmunoResearch) in 1:200 in PBS containing 1% BSA and 4',6-diamidino-2-phenylindole (DAPI) nuclear stain was added and incubated for 1 hour at 37°C. After 4 washes with PBS, 150 µL of PBS was added to each well, and microscopy was performed using a Leica DMi8 with 20X objectives. The same microscopy settings were used

for all images. ImageJ version 2.1.0. was used for processing, and all images were linearly stretched equally. The concentration of each antibody analyzed (3.3 nM) corresponded to the IC_{50} of the mIgA1 antibody.

Surface plasmon resonance (SPR).

Antibody binding properties were analyzed at 25 °C using a Biacore 8K instrument (GE Healthcare) with 10 mM HEPES pH 7.4, 150 mM NaCl, 3 mM EDTA, and 0.005% Tween-20 as running buffer. SARS-CoV-2 S-trimers (2, 7, 35 and 70 nM) were immobilized on the surface of a CM5 chip (Cytiva) by standard amine coupling. Increasing concentrations of antibodies (3.125, 6.25, 12.5 25, and 50 nM) were injected at a single-cycle kinetics setting (association time: 180 sec, flow rate: 30 µL/min), and dissociation was followed for 10 or 30 mins. Analyte responses were corrected for nonspecific binding and buffer responses. Curve fitting and data analysis were performed with Biacore™ Insight Evaluation Software v.2.0.15.12933. The fitting of K_{on} and K_{off} was performed separately using a 1:1 binding model for K_{on} and a 1:1 dissociation model for K_{off}.

Computational modelling.

Computational structure modelling was performed based on the binding of 01A05 to the RBD of variants (Alpha, Beta, Delta, and Omicron) or through previously published information describing docking (rmAb23) (12) or crystallization studies (Protein Data Bank (PDB) ID: 7CH4 for DXP-604 and PDB ID: 7V2A for XG014) (7, 10).

The 01A05 variable fragment was modelled according to the canonical structure method with the program RosettaAntibody (16) as previously described (17). Docking was performed using RosettaDock v3.1 as previously described (18). In summary, the 01A05 model was

docked to the WT RBD experimental structure (PDB ID: 6m17). Among the thousands of computationally generated complexes, the decoy in better agreement with experimental data (competition with hACE2 and differential neutralization activity against SARS-CoV-2 variants) was selected and further refined by computational docking.

The selected models of 01A05 and rmAb23 were subjected to a 350 ns molecular dynamics (MD) simulation to adjust the local geometry and verify that the structure was energetically stable. MD was performed with GROMACS (19). The system was initially set up and equilibrated through standard MD protocols: proteins were centered in a triclinic box, 0.2 nm from the edge, filled with SPCE water model and 0.15 m $Na^{+}Cl^{-}$ ions using the AMBER99SB-ILDN protein force field. Energy minimization was performed to allow the ions to achieve a stable conformation. Temperature and pressure equilibration steps, respectively at 310 K and 1 Bar, of 100 ps each were completed before performing the full MD simulations with the abovementioned force field. MD trajectory files were analyzed after removal of the periodic boundary conditions. The stability of each simulated complex was verified by root mean square deviation and visual analysis.

The structures of monomeric IgG, mIgA1 and dIgA1 DXP-604 bound to two SARS-CoV-2 S trimers were built using PyMOL software (The PyMOL Molecular Graphics System, Version 2.0 Schrödinger, LLC).

Tracking antibody bio-distribution in mice.

DXP-604 dIgA1 antibodies were conjugated with Alexa Fluor 647 dye for *in vivo* and *ex vivo* imaging studies. In brief, 6.26 mg/ml solution of DXP-604 dIgA1 in PBS was incubated with Alexa Fluor 647 succinimidyl ester (AF647-NHS, Invitrogen) in the presence of 1M sodium

bicarbonate buffer using a molar ratio of 1:10 protein to fluorescent probe at room temperature for 1 h. Unreacted dye was removed by dialysis and the labelled antibody was washed in PBS. All procedures were performed under dimmed light.

The ICR mice (8–10 weeks, male, Beijing Vital River Laboratory Animal Technology Co., Ltd.) were injected with ketamine hydrochloride (8 mg/100 g) and placed in a supine position. Subsequently, the mice were intranasally administered Alexa Fluor 647-labelled DXP-604 dIgA1 to both nostrils using a fine pipette tip, in a volume of 50 µL, to achieve a final antibody dose of 60 µg per mouse. The mice were imaged while alive using an Ivis Lumina Xr (Caliper Life Sciences), both before and at predetermined time points (2 min, 16 min, 2 h and 6 h) following the administration of antibodies. The imaging parameters included fluorescence excitation at a wavelength of 679 nm, emission at a wavelength of 702 nm, and an auto-exposure setting. At different time points (2, 6, 12, 24, 48 and 72 h) following the administration of antibodies, mice were euthanized, and the heart, lungs (with a section of trachea), liver, spleen, kidney, and nasal cavity were excised and imaged. Regions of interest (ROIs) were drawn and average radiant efficiency (p/s/cm^2/sr)/(µW/cm^2) was measured. This parameter represents the sum of the radiance from each pixel inside the ROI divided by the number of pixels. All images were processed using Living Image software and the same fluorescence threshold was applied for mice comparison. The procedures were approved by Institutional Animal Care and Use Committee, Kunming Institute of Zoology, Chinese Academy of Sciences (IACUC-RE-2023-07-007).

Antibody protection in an animal model.

The animal study was performed in an animal biosafety level 3 (ABSL3) facility using HEPA-filtered isolators. All animal procedures were approved by the Institute of Laboratory

Animal Sciences (ILAS), Chinese Academy of Medical Sciences (CAMS) and Peking Union Medical College (PUMC) (BLL22007).

SARS-CoV-2/human/CHN/GD-5/2022 (Omicron BA.5, GenBank: OP678016) was provided by Guangdong Provincial Center for Disease Prevention and Control. The virus was produced with VERO E6 cells, and the SARS-CoV-2 titer was determined by the $TCID_{50}$ method.

Specific-pathogen-free (SPF) ICR-hACE2 mice, 8-10 weeks old (18-32 g), were provided by the Institute of Medical Experimental Animals, Chinese Academy of Medical Sciences. hACE2 mice were randomly divided into 4 groups with three mice in each group. The mice were anaesthetized using intraperitoneal injection of 2.5% tribromoethanol (Avertin) for both viral inoculation and antibody administration. The animals were inoculated intranasally with 50 µl of authentic SARS-CoV-2 Omicron BA.5 (10^5 TCID50/ml) (20, 21). The prophylactic and therapeutic doses of dimeric IgA were established based on prior studies involving intranasal administration of IgM for protection against SARS-CoV-2 (22). For therapeutic treatment, the mice were administered 60 µg DXP-604 dIgA1 using a nasal drop (50 µl), 2 hours after viral challenge, and for prophylactic treatments, 40 or 60 µg DXP-604 dIgA1 was administered 4 hours prior to challenge. The negative control group received PBS only. The infected mice were observed daily to record symptoms. The animals were euthanized by exsanguination at 3 days post-infection while under deep anaesthesia. Tissue specimens, including samples from the lung and trachea, were collected for quantification of viral load.

Viral load analysis was performed by qRT-PCR. Lung and trachea homogenates were prepared by using an electric homogenizer. The total RNA of the lungs and trachea was extracted with the RNeasy Mini Kit (Qiagen). Reverse transcription was processed with the PrimerScript RT Reagent Kit (TaKaRa) according to the manufacturers' instructions. qRT-

PCR reactions were performed using the PowerUp SYBR Green Master Mix Kit (Applied Biosystems), according to the following cycling protocol: 50°C 2 min, 95°C 2 min, followed by 95°C 15 s, 60°C 30 s for 40 cycles, and then by the melting curve analysis: 95°C 15 s, 60°C 1 min, 95°C 45 s. Forward primer 5′-TCGTTTCGGAAGAGACAGGT-3′ and reverse primer 5′-GCGCAGTAAGGATGGCTAGT-3′ were used in qRT-PCR. Standard curves were constructed by using 10-fold serial dilutions of recombinant plasmids with known copy numbers (from 1.47×10^9 to 1.47×10^1 copies/μl).

Quantification and statistical analysis.

A two-sided Mann–Whitney U test was performed for comparisons of anti-SARS-CoV-2 antibody levels between groups. A Wilcoxon signed-rank test was used for comparison of paired samples. Correlation analysis between antibody levels was performed using Spearman's rank correlation. A two-sided t-test was used to compare the viral load in the mouse model. All analyses and data plotting were performed with GraphPad 7.04. A p value less than 0.05 was considered to be statistically significant.

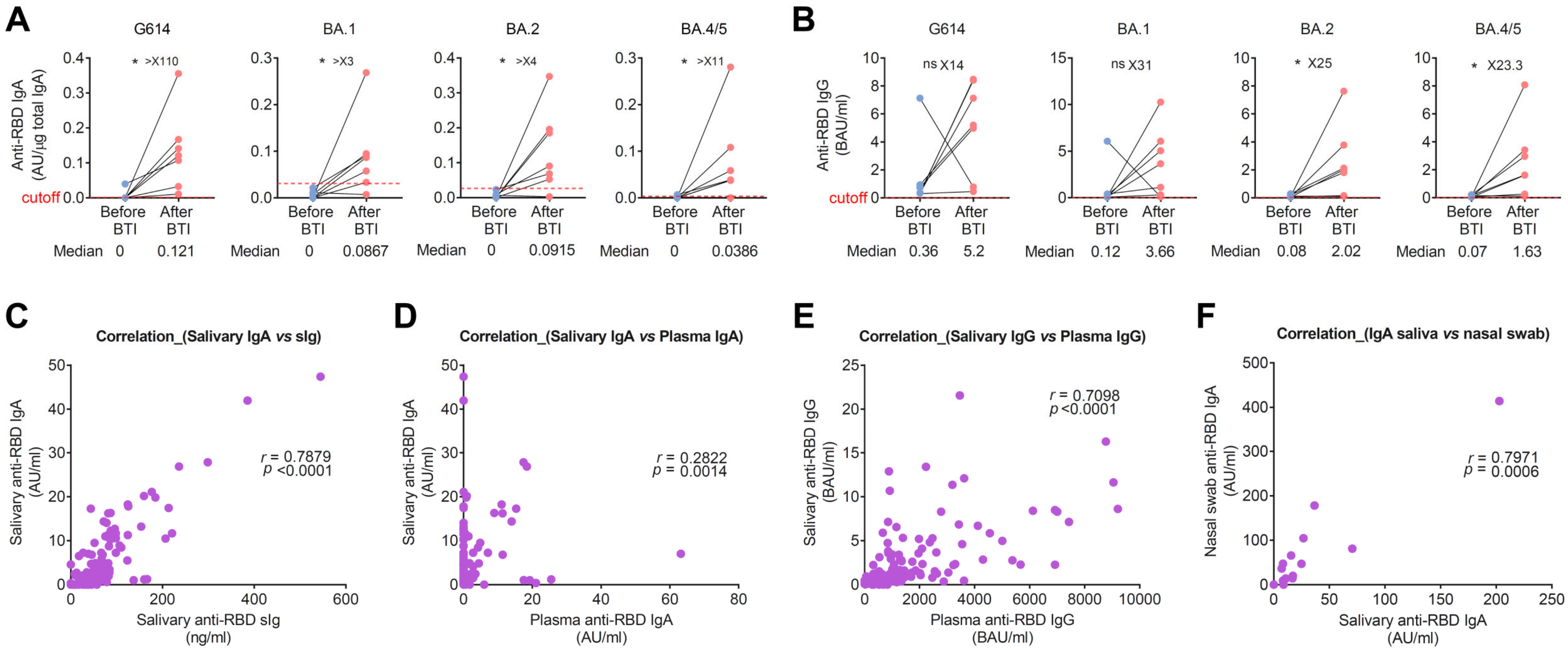

Fig. S1. Salivary anti-RBD IgA antibodies correlate with salivary anti-RBD secretory immunoglobulins (sIg) and increase after breakthrough infection. (*A* and *B*) Salivary anti-RBD IgA (*A*) and IgG (*B*) antibodies against G614 and Omicron variants BA.1, BA.2 and BA.4/5 in paired samples before and after breakthrough infection (BTI) in mRNA-vaccinated individuals (n =7). The number of fold differences in anti-RBD antibody titers are indicated. A Wilcoxon paired-sample signed-rank test was used. *$P < 0.05$. Samples were collected 14-198 days after the second dose of mRNA vaccine and 14-38 days after breakthrough infection (*C*-*F*) Correlation between salivary anti-RBD IgA and salivary anti-RBD secretory immunoglobulin (sIg) (*C*), salivary anti-RBD IgA and plasma anti-RBD IgA (*D*), salivary anti-RBD IgG and plasma anti-RBD IgG antibodies (*E*), and nasal anti-RBD IgA and salivary anti-RBD IgA (*F*). Correlation analysis was performed using Spearman's rank correlation. In *F*, saliva and nasal fluid samples were collected 18 to 19 months after their last vaccine dose or 3 to 20 months after experiencing BTI. Statistically significant if $P < 0.05$.

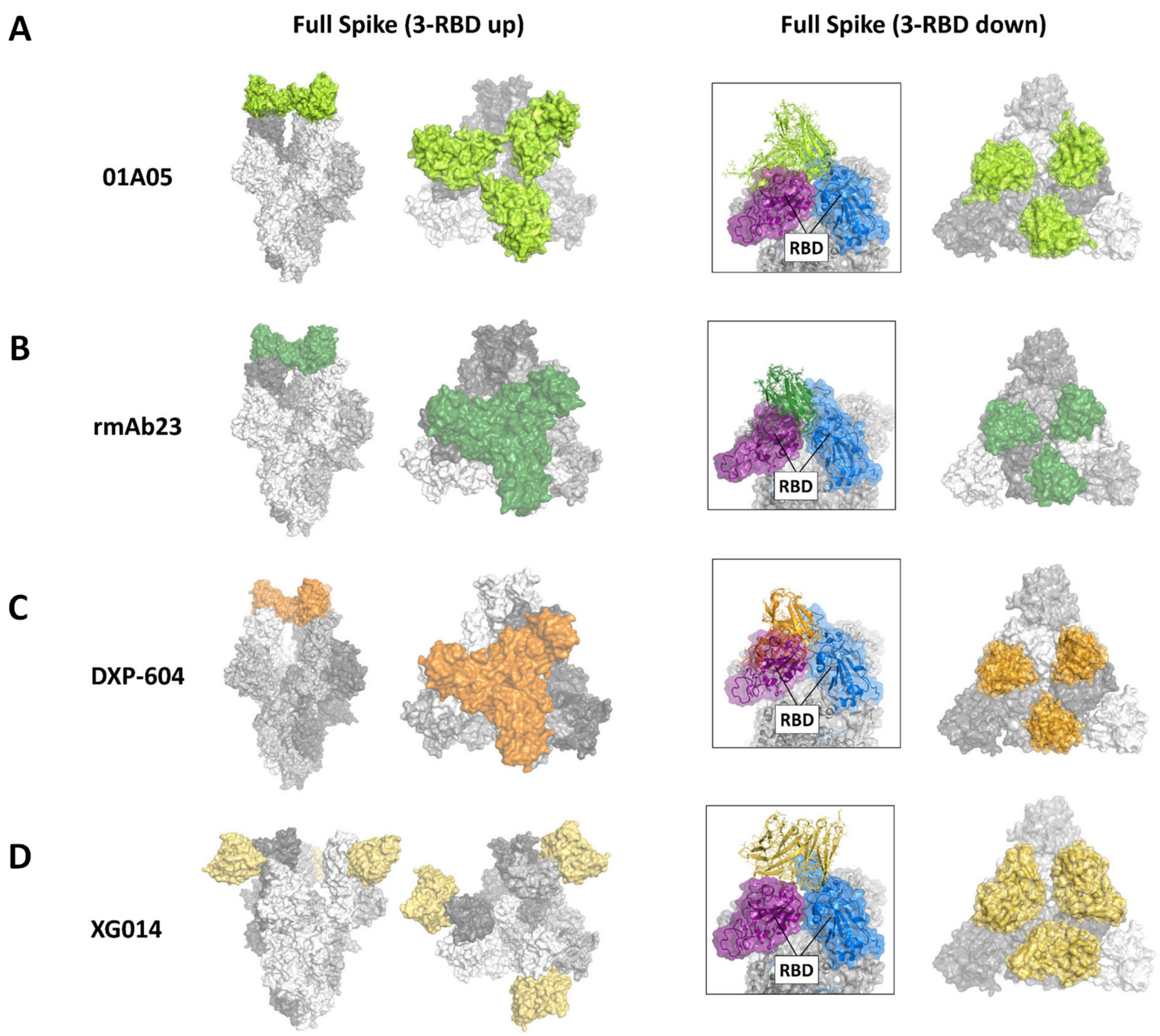

Fig. S2. Computational simulations predicted the binding of neutralizing antibodies to RBDs in the S-trimer. (*A-D*) Probability of 01A05 (*A*), rmAb23 (*B*), DXP-604 (*C*) and XG014 (*D*) binding to all three receptor-binding domains (RBDs) in the S-trimer (full S protein) in the up (3-RBD up) or down (3-RBD down) conformation. Three 01A05 can simultaneously bind the RBD on the S-trimer in the up conformation (3-RBD up) (*A*). One single rmAb23 Fab bound to the S-trimer with the 3-RBD in the up position (*B*). A single DXP-604 Fab bound to the S-trimer with the 3-RBD in the up position can prevent binding of ACE2 to all three S monomers and prevent the binding of other Fabs to S monomers (*C*). The epitopes of 01A05, mAb23 and DXP-604 are inaccessible on trimeric S 3-RBD down (*A*-*C*) because the antibodies interfere with the RBDs of the adjacent S monomer (in purple). Three XG014 Fabs can bind all three RBDs in the "down" conformation (3-RBD down) and should be able to bind the RBD in the up position (*D*).

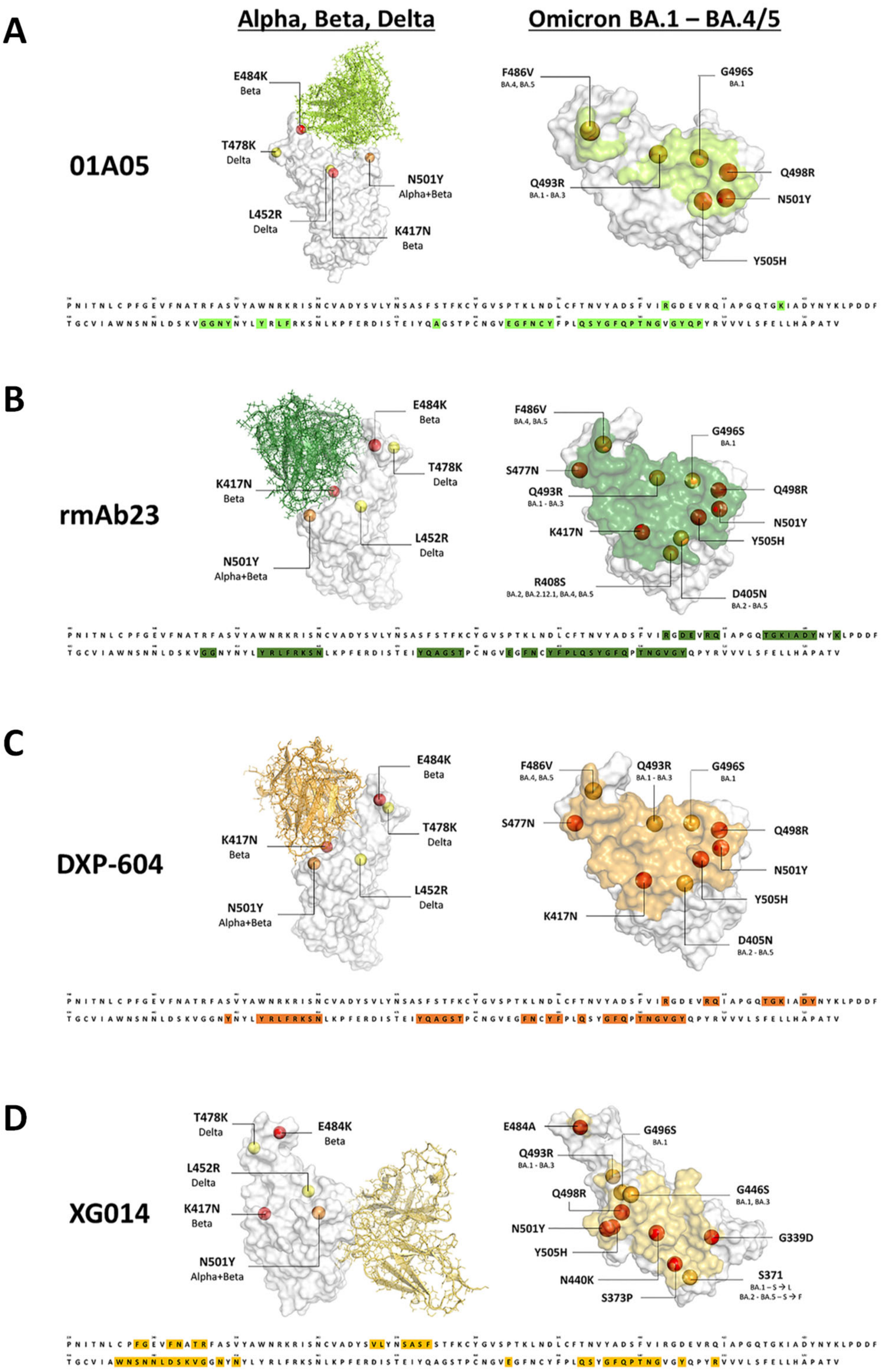

Fig. S3. Computational simulations predicted antibody binding to the RBDs of variants of concern (VOCs). (*A-D*) Docking model of 01A05 (*A*), rmAb23 (*B*), DXP-604 (*C*) and XG014 (*D*) to RBD of Alpha, Beta and Delta (left panel) and Omicron BA.1, BA.2 and BA.4/5 (right panel), including positions of mutations. Left panel: Spheres represent mutations present in Delta (yellow), Beta (red), or both Alpha and Beta (orange). Right panel: Spheres represent mutations in both BA.1, BA.2, BA.4, and BA.5 (red) or only some of the Omicron variants (orange), as indicated below the mutation. The sequence below each model shows the RBD epitope residues (highlighted) that make contact with an antibody.

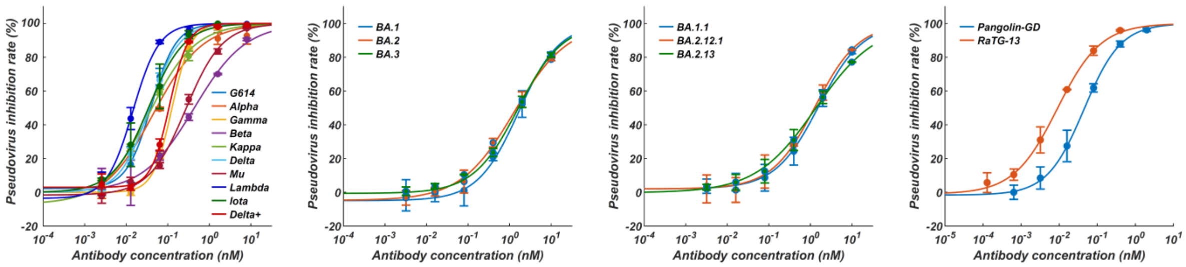

Pseudovirus neutralization IC50 (nM)

Variants	Alpha	Beta	Gamma	Delta	Kappa	Delta plus	Mu	Lambda	Lota
	B.1.1.7	B.1.351	P.1	B.1.617.2	B.1.617.1	AY.1	B.1.621	C.37	B.1.621
IC50 (nM)	0.05	0.43	0.13	0.03	0.03	0.11	0.27	0.01	0.03

Variants	Omicron	Omicron	Omicron	Omicron	Omicron	Omicron	RaTG13	Pangolin
	BA.1	BA.2	BA.3	BA.1.1	BA.2.12.1	BA.2.13		
IC50 (nM)	1.53	1.2	1.6	1.6	1.33	1.33	0.01	0.05

IC50 (nM)
0-0.01
0.01-0.1
0.1-1
>1

Fig. S4. DXP-604 broadly and potently neutralized SARS-CoV-2 variants. (*A*-*D*) DXP-604 neutralization of SARS-CoV-2 variant S-pseudotyped VSV, including D614G, Alpha (B.1.1.7), Beta 453 (B.1.351), Gamma (P.1), Delta (B.1.617.2), 454 Kappa (B.1.617.1), Delta plus (AY.1), Mu (B.1.621), Lambda (C.37) and Iota (B.1.526) (*A*); BA.1, BA.2 and BA.3 (*B*); BA.1/BA.2 subvariants BA.1.1 (BA.1+R346K), BA.2.12.1 (BA.1+L452Q+S704L) and BA.2.13 (BA.1+L452M) (*C*); and clade 1b SARS-CoV-2 related sarbecoviruses (RaTG13 and Pangolin-GD) (*D*). Each experiment was performed in triplicate and the mean neutralization (%) values are presented. The IC_{50} values are indicated in the table.

A 01A05

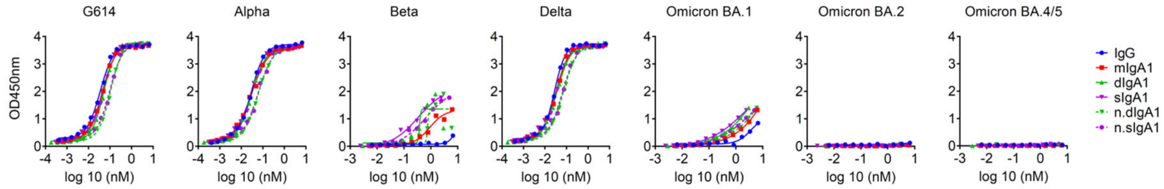

Binding EC50 (nM)

	G614	Alpha	Beta	Delta	Omicron BA.1	Omicron BA.2	Omicron BA.4/5
IgG	0.04	0.03	>6.67	0.03	>6.67	>6.67	>6.67
mIgA1	0.04	0.03	>6.25	0.03	>6.25	>6.25	>6.25
dIgA1	0.05	0.03	>2.99	0.03	>2.99	>2.99	>2.99
sIgA1	0.05	0.03	>2.44	0.03	>2.44	>2.44	>2.44
n.dIgA1	0.10	0.06	>5.98	0.07	>5.98	>5.98	>5.98
n.sIgA1	0.10	0.06	>4.88	0.07	>4.88	>4.88	>4.88

EC50 (nM): 0-0.5; 0.5-2; 2-5; >5

Fold change to IgG

	G614	Alpha	Beta	Delta	Omicron BA.1	Omicron BA.2	Omicron BA.4/5
mIgA1	0.82	0.96	–	0.25	–	–	–
dIgA1	0.68	0.94	–	0.30	–	–	–
sIgA1	0.73	0.95	–	0.26	–	–	–
n.dIgA1	0.34	0.47	–	0.15	–	–	–
n.sIgA1	0.37	0.48	–	0.13	–	–	–

Fold change: <1; 1-5; 5-10; 10-50; >50

B rmAb23

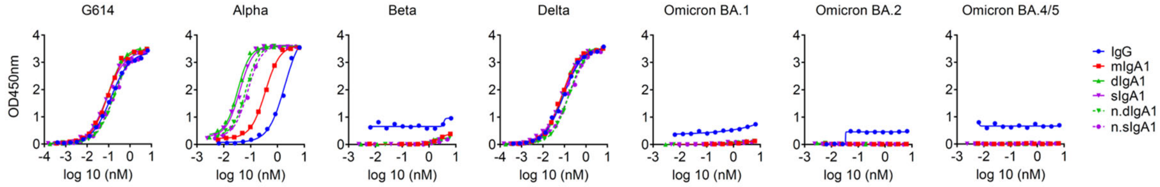

Binding EC50 (nM)

	G614	Alpha	Beta	Delta	Omicron BA.1	Omicron BA.2	Omicron BA.4/5
IgG	0.13	1.89	>6.67	0.09	>6.67	>6.67	>6.67
mIgA1	0.09	0.35	>6.25	0.07	>6.25	>6.25	>6.25
dIgA1	0.09	0.03	>2.99	0.08	>2.99	>2.99	>2.99
sIgA1	0.08	0.04	>2.44	0.08	>2.44	>2.44	>2.44
n.dIgA1	0.18	0.07	>5.98	0.16	>5.98	>5.98	>5.98
n.sIgA1	0.17	0.08	>4.88	0.16	>4.88	>4.88	>4.88

EC50 (nM): 0-0.5; 0.5-2; 2-5; >5

Fold change to IgG

	G614	Alpha	Beta	Delta	Omicron BA.1	Omicron BA.2	Omicron BA.4/5
mIgA1	1.42	5.38	–	1.25	–	–	–
dIgA1	1.41	57.89	–	1.17	–	–	–
sIgA1	1.53	48.58	–	1.13	–	–	–
n.dIgA1	0.71	28.95	–	0.59	–	–	–
n.sIgA1	0.77	24.29	–	0.57	–	–	–

Fold change: <1; 1-5; 5-10; 10-50; >50

C XG014

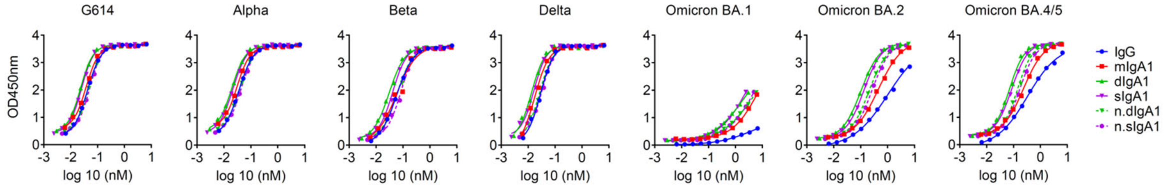

Binding EC50 (nM)

	G614	Alpha	Beta	Delta	Omicron BA.1	Omicron BA.2	Omicron BA.4/5
IgG	0.04	0.04	0.06	0.03	>6.67	1.02	0.33
mIgA1	0.04	0.03	0.06	0.02	>6.25	0.45	0.24
dIgA1	0.02	0.02	0.03	0.01	>2.99	0.10	0.07
sIgA1	0.03	0.02	0.04	0.02	>2.44	0.13	0.09
n.dIgA1	0.05	0.04	0.06	0.03	>5.98	0.21	0.14
n.sIgA1	0.05	0.04	0.08	0.03	>4.88	0.26	0.17

EC50 (nM): 0-0.5; 0.5-2; 2-5; >5

Fold change to IgG

	G614	Alpha	Beta	Delta	Omicron BA.1	Omicron BA.2	Omicron BA.4/5
mIgA1	1.12	1.17	0.99	1.65	–	2.25	1.41
dIgA1	1.81	1.83	1.89	2.16	–	9.89	4.71
sIgA1	1.66	1.67	1.39	1.75	–	7.71	3.88
n.dIgA1	0.91	0.92	0.95	1.08	–	4.95	2.36
n.sIgA1	0.83	0.84	0.70	0.88	–	3.86	1.94

Fold change: <1; 1-5; 5-10; 10-50; >50

Fig. S5. Dimeric and secretory IgA1 enhanced binding activity against variants of concern (VOCs). (*A*-*C*) Binding of IgG and IgA1 antibodies 01A05 (*A*), rmAb23 (*B*), and XG014 (*C*) to the RBD of G614 and VOCs (Alpha, Beta, Delta and Omicron), as determined by ELISAs. The EC_{50} and fold-change differences between the IgG and IgA antibody forms are indicated. n.dIgA and n.sIgA represent normalized values according to the number of binding sites.

A **01A05**

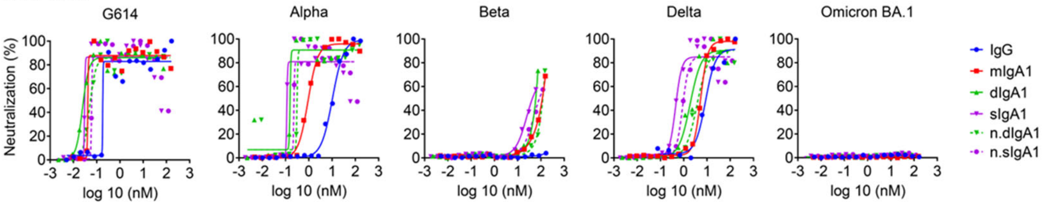

Neutralization IC50 (nM)

	G614	Alpha	Beta	Delta	Omicron BA.1
IgG	0.18	10.41	>167	8.62	>167
mIgA1	0.04	0.93	>156	5.38	>156
dIgA1	0.02	0.16	>74	1.90	>74
sIgA1	0.03	0.11	24.56	0.45	>61
n.dIgA1	0.05	0.31	>148	3.80	>128
n.sIgA1	0.06	0.23	49.12	0.90	>122

IC50 (nM): 0-0.5, 0.5-2, 2-5, >5

Fold change to IgG

	G614	Alpha	Beta	Delta	Omicron BA.1
mIgA1	4.41	11.22	–	1.60	–
dIgA1	7.45	66.39	–	4.53	–
sIgA1	6.04	91.40	>6.80	19.18	–
n.dIgA1	3.73	33.20	–	2.27	–
n.sIgA1	3.02	45.70	>3.4	9.59	–

Fold change: <1, 1-5, 5-10, 10-50, >50

B **rmAb23**

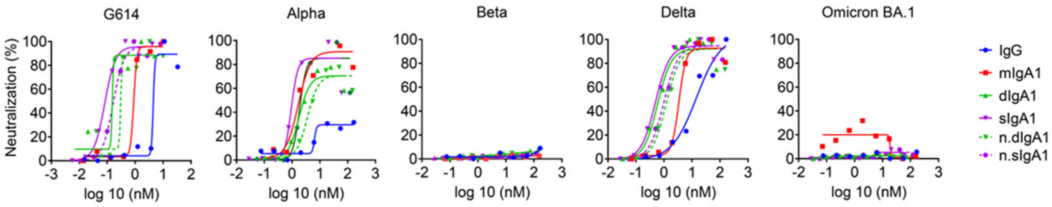

Neutralization IC50 (nM)

	G614	Alpha	Beta	Delta	Omicron BA.1
IgG	4.44	>167	>167	14.45	>167
mIgA1	0.89	1.66	>156	3.42	>156
dIgA1	0.15	1.79	>74	0.59	>74
sIgA1	0.08	0.93	>61	0.49	>61
n.dIgA1	0.30	3.57	>148	1.18	>128
n.sIgA1	0.16	1.87	>112	0.98	>112

IC50 (nM): 0-0.5, 0.5-2, 2-5, >5

Fold change to IgG

	G614	Alpha	Beta	Delta	Omicron BA.1
mIgA1	4.99	>100.6	–	4.23	–
dIgA1	29.21	>93.56	–	24.47	–
sIgA1	54.44	>178.82	–	29.35	–
n.dIgA1	14.61	>46.78	–	12.24	–
n.sIgA1	27.22	>89.41	–	14.68	–

Fold change: <1, 1-5, 5-10, 10-50, >50

C **XG014**

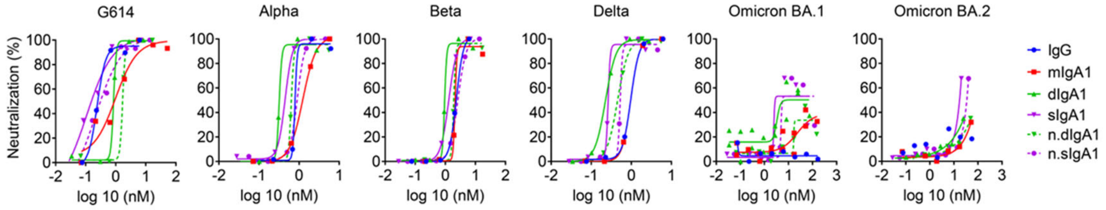

Neutralization IC50 (nM)

	G614	Alpha	Beta	Delta	Omicron BA.1	Omicron BA.2
IgG	0.24	0.79	2.34	0.97	>167	>167
mIgA1	0.98	1.24	2.10	0.97	23.32	>156
dIgA1	0.85	0.32	0.95	0.23	3.29	>74
sIgA1	0.11	0.45	1.31	0.26	4.44	>61
n.dIgA1	1.70	0.64	1.89	0.45	6.57	>148
n.sIgA1	0.22	0.90	2.62	0.53	8.89	>121

IC50 (nM): 0-0.5, 0.5-2, 2-5, >5

Fold change to IgG

	G614	Alpha	Beta	Delta	Omicron BA.1	Omicron BA.2
mIgA1	0.24	0.64	1.11	1.01	>7.16	–
dIgA1	0.28	2.48	2.47	4.28	>50.84	–
sIgA1	2.21	1.76	1.78	3.69	>37.58	–
n.dIgA1	0.14	1.24	1.24	2.14	>25.42	–
n.sIgA1	1.11	0.88	0.89	1.85	>18.79	–

Fold change: <1, 1-5, 5-10, 10-50, >50

Fig. S6. Dimeric and secretory IgA1 enhanced neutralization activity against variants of concern (VOCs). (*A*-*C*) Neutralization activity of IgG and IgA1 antibodies 01A05 (*A*), rmAb23 (*B*), and XG014 (*C*) against G614 and VOCs (Alpha, Beta, Delta and Omicron) as determined via microneutralization assay. Each experiment was performed in triplicate, and the mean neutralization (%) values are presented. The IC_{50} and fold-change differences between the IgG and IgA antibody forms are indicated. n.dIgA and n.sIgA represent normalized values according to the number of binding sites.

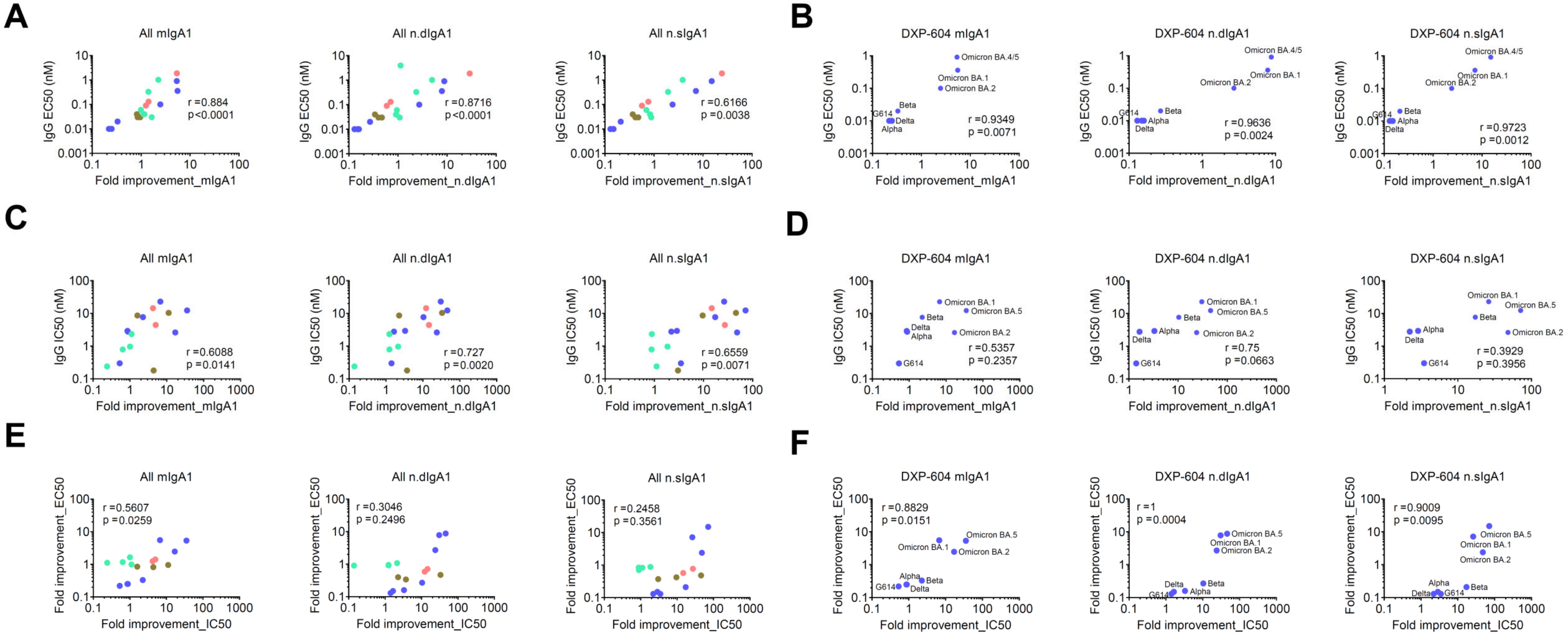

Fig. S7. Increased neutralization after switching to IgA1 and dimerization was associated with increased RBD binding. (*A-D*) Correlation between EC_{50} or IC_{50} of all four IgG antibodies (01A05, rmAb23, DXP-604, and XG014) (*A* and *C*) or DXP-604 only (*B* and *D*) with increased binding fold changes (*A* and *B*) or neutralization fold changes (*C* and *D*) after conversion to monomeric (mIgA1), dimeric (dIgA1) or secretory IgA1 (sIgA1). (*E* and *F*) Correlation between the fold change increase in RBD binding and neutralization activity for all four IgG antibodies (*E*) or DXP-604 only (*F*) after conversion to mIgA1, dIgA1 and sIgA1. The EC50 and IC50 values of n.dIgA1 and n.sIgA1, which were normalized based on the number of binding sites, were used for correlation analysis.

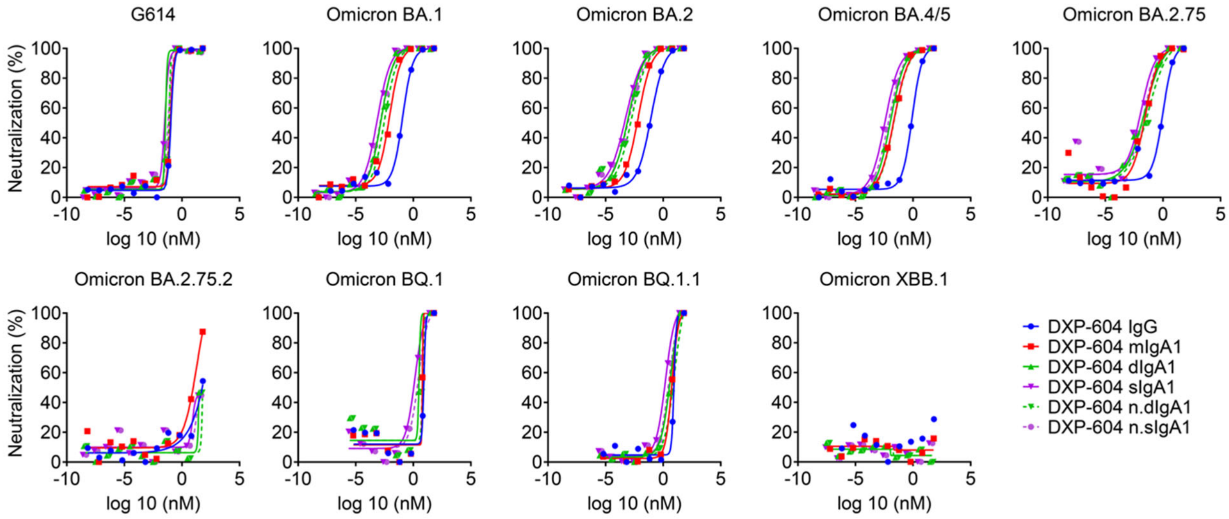

Pseudovirus neutralization IC50 (nM)

	G614	Omicron BA.1	Omicron BA.2	Omicron BA.4/5	Omicron BA.2.75	Omicron BA.2.75.2	Omicron BQ.1	Omicron BQ.1.1	Omicron XBB.1
IgG	0.1129	0.1205	0.0909	0.8355	1.0160	>66.67	8.4500	8.7530	>66.67
mIgA1	0.1007	0.0089	0.0069	0.0221	0.0304	>62.5	6.2280	5.7670	>62.5
dIgA1	0.0374	0.0017	0.0009	0.0110	0.0275	>29.85	3.2010	4.8770	>29.85
sIgA1	0.0327	0.0008	0.0006	0.0038	0.0130	>24.39	1.4130	1.5910	>24.39
n.dIgA1	0.0748	0.0034	0.0018	0.0220	0.0550	>59.70	6.4020	9.7540	>59.70
n.sIgA1	0.0654	0.0016	0.0012	0.0076	0.0261	>48.78	2.8260	3.1820	>48.78

IC50 (nM): <0.001; 0.001-0.01; 0.01-0.1; >0.1

Fold change to IgG

	G614	Omicron BA.1	Omicron BA.2	Omicron BA.4/5	Omicron BA.2.75	Omicron BA.2.75.2	Omicron BQ.1	Omicron BQ.1.1	Omicron XBB.1
mIgA1	1.12	13.54	13.17	37.81	33.43	–	1.36	1.52	–
dIgA1	3.02	70.88	101.00	75.95	36.97	–	2.64	1.79	–
sIgA1	3.45	150.63	151.50	219.87	77.97	–	5.98	5.50	–
n.dIgA1	1.51	35.44	50.50	37.98	18.49	–	1.32	0.90	–
n.sIgA1	1.73	75.31	75.75	109.93	38.99	–	2.99	2.75	–

Fold change: <1; 1-10; 10-50; 50-100; >100

Fig. S8. DXP-604 dimeric and secretory IgA1 enhanced neutralization activity against emerging Omicron subvariants. DXP-604 neutralization against SARS-CoV-2 S pseudotyped HIV-1-based viruses, including G614, Omicron BA.1, BA.2, BA.4/5 and subvariants (BA.2.75, BA.2.75.2, BQ.1, BQ.1.1 and XBB.1). Each experiment was performed in duplicate, and the mean neutralization (%) values are presented. The IC_{50} and fold-change differences between the IgG and IgA antibody forms are indicated. n.dIgA and n.sIgA represent the normalized values based on the number of binding sites.

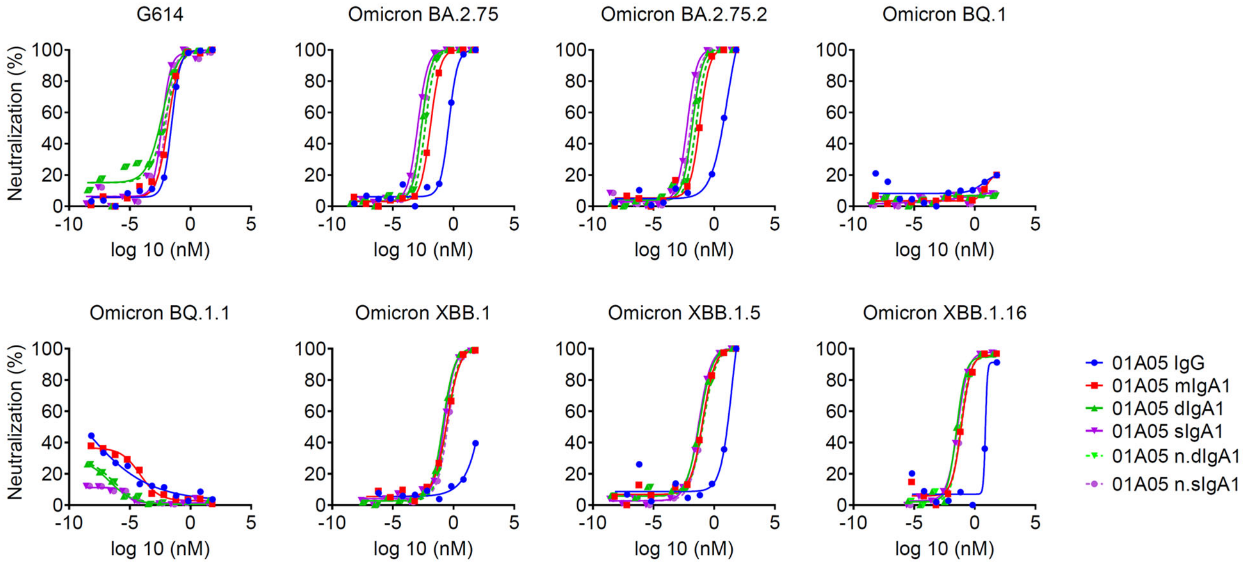

Pseudovirus neutralization IC50 (nM)

	G614	Omicron BA.2.75	Omicron BA.2.75.2	Omicron BQ.1	Omicron BQ.1.1	Omicron XBB.1	Omicron XBB.1.5	Omicron XBB.1.16
IgG	0.0280	0.4195	12.0800	>66.67	>66.67	>66.67	30.4800	7.5180
mIgA1	0.0140	0.0127	0.0671	>62.5	>62.5	0.3000	0.1154	0.0791
dIgA1	0.0048	0.0024	0.0165	>29.85	>29.85	0.1347	0.0658	0.0314
sIgA1	0.0045	0.0012	0.0060	>24.39	>24.39	0.1754	0.0528	0.0381
n.dIgA1	0.0096	0.0049	0.0330	>59.70	>59.70	0.2694	0.1316	0.0628
n.sIgA1	0.0091	0.0023	0.0119	>48.78	>48.78	0.3508	0.1056	0.0762

IC50 (nM): <0.001; 0.001-0.01; 0.01-0.1; >0.1

Fold change to IgG

	G614	Omicron BA.2.75	Omicron BA.2.75.2	Omicron BQ.1	Omicron BQ.1.1	Omicron XBB.1	Omicron XBB.1.5	Omicron XBB.1.16
mIgA1	2.00	32.98	180.08	–	–	>222.23	264.12	95.04
dIgA1	5.85	172.99	733.01	–	–	>494.95	463.22	239.43
sIgA1	6.19	364.78	2023.45	–	–	>380.10	577.27	197.32
n.dIgA1	2.93	86.49	366.50	–	–	>247.48	231.61	119.71
n.sIgA1	3.09	182.39	1011.73	–	–	>190.05	288.64	98.66

Fold change: <1; 1-10; 10-50; 50-100; >100

Fig. S9. 01A05 dimeric and secretory IgA1 enhanced neutralization activity against emerging Omicron subvariants. 01A05 neutralization against SARS-CoV-2 S pseudotyped HIV-1-based viruses, including G614 and Omicron subvariants (BA.2.75, BA.2.75.2, BQ.1, BQ.1.1, XBB.1, XBB.1.5, and XBB.1.16). Each experiment was performed in duplicate, and the mean neutralization (%) values are presented. The IC_{50} and fold-change differences between the IgG and IgA antibody forms are indicated. n.dIgA and n.sIgA represent the normalized values based on the number of binding sites.

Table S1. Demographic data of vaccinated individuals.

Groups	Number	Male/Female	Median age (IQR)[a], years	Median sampling day after vaccination (IQR)
Before vaccination	7	3/4	37 (28-38)	
Inactivated vaccine				
2nd dose	5	2/3	29 (28-32)	35 (16-70)
3rd dose	6	2/4	27.5 (26-35)	68.5 (38-91)
mRNA vaccine				
1st dose	18	7/11	35.5 (27-40)	17.5 (14-21)
2nd dose	36	15/21	38.5 (28-52)	28 (17-52)
3rd dose	15	3/12	34 (29-53)	19 (15-28)
Heterologous vaccine: Inactivated + 1 dose mRNA vaccine	13	5/8	28 (27-30)	27 (19-36)
Infected + mRNA vaccine	10	6/4	43 (32-52)	30 (17-37)
BTI	19	6/13	30 (27-52)	19 (14-31)

[a] IQR: Interquartile range.

[b] Breakthrough infection after two or three doses of either inactivated or mRNA vaccine or a combination of both during the Omicron BA.1 wave. Data from a subset of the samples from donors receiving the mRNA vaccine (2nd dose, n=34; and 3rd doses, n=15), those who experienced BTI after the mRNA vaccine (n=12), or negative control (n=7) were previously described (1) and included for comparison.

Table S2. Comparison of DPX604-IgG and IgA1 neutralization activity with commercially available antibodies and antibodies described in the literature.

Antibodies	Neutralization activity (IC50 normalized based on the number of binding sites)															References[a]
	Pseudovirus (pM)							Authentic virus (nM)								
	G614 or WT	Alpha	Beta	Delta	Omicron BA.1	Omicron BA.2	Omicron BA.4/5	G614	Alpha	Beta	Delta	Omicron BA.1	Omicron BA.2	Omicron BA.4	Omicron BA.5	
**DXP-604 IgG**	112.9	ND[b]	ND	ND	120.5	90.9	835.5	0.30	2.93	7.71	2.78	22.93	2.63	ND	12.36	This paper
**DXP -604 mIgA1**	100.7	ND	ND	ND	8.90	6.9	22.1	0.56	3.41	3.42	3.15	3.43	0.16	ND	0.35	This paper
**DXP -604 dIgA1**	74.8	ND	ND	ND	3.4	1.8	22.0	0.21	0.88	0.74	1.69	0.75	0.11	ND	0.27	This paper
**DXP -604 sIgA1**	65.4	ND	ND	ND	1.6	1.2	7.6	0.09	1.02	0.45	1.26	0.87	0.05	ND	0.17	This paper
LY-CoV1404 (bebtelovimab, Eli Lilly)	4	ND	14	ND	4	6	6	0.070	0.027	0.047	0.053	0.107	ND	0.08	0.10	Cao et al. 2022 (23) Westendorf et al. 2022 (24) Turelli et al. 2022 (25)
COV2-2130	16.67	ND	ND	ND	20 050	42	153	ND	ND	ND	ND	ND	ND	ND	ND	Cao et al. 2022 (23)
SA58(BD55-5840)	6	ND	ND	ND	29.33	80	26	ND	ND	ND	ND	ND	ND	ND	ND	Cao et al. 2022 (23)
SA55(BD55-5514)	73.3	ND	ND	ND	11.33	48	33.3	ND	ND	ND	ND	ND	ND	ND	ND	Cao et al. 2022 (23)
REGN10987 (Regeneron)	38	>66 670	13.33	33.33	> 66 670	3 930	3 470	ND	ND	ND	ND	ND	ND	ND	ND	Cao et al 2022 (23)
S309	493.33	ND	ND	ND	2 410	6 120	5 280	1.23	ND	ND	ND	3.01	39.23	ND	ND	Cao et al. 2022 (23)

																Case et al. 2022 (23)
REGN10933+ REGN10987 (Regeneron)	33.33	ND	ND	ND	>66 670	5 470	4 730		ND	ND	ND	ND	ND	ND	ND	Cao et al. 2022 (23)
AZD1061 (Astra Zeneca)	13.33	ND	ND	ND	2 050	53.33	100	0.197	ND	ND	ND	40.52	0.21	0.81	0.95	Case et al. 2022 (26) Tuekprakhon et al. 2022 (27) Turelli et al. 2022 (25)
AZD7442 (Astra Zeneca) (Tixagevimab+ Cilgavimab)	6.67	ND	ND	ND	1 550	53.33	433	0.043	ND	ND	ND	1.11	0.24	ND	ND	Case et al 2022 (26) Tuekprakhon et al. 2022 (27)

Dataset S1 (separate file): Dataset used to create the figures in the paper.

SI References

1. Zuo, F., Marcotte, H., Hammarström, L., Pan-Hammarström, Q., Mucosal IgA against SARS-CoV-2 Omicron Infection. *N. Engl. J. Med.* **387**, e55 (2022).

2. F. Zuo, *et al.*, Heterologous immunization with inactivated vaccine followed by mRNA-booster elicits strong immunity against SARS-CoV-2 Omicron variant. *Nat. Commun.* **13**, 2670 (2022).

3. N. Sherina, *et al.*, Persistence of SARS-CoV-2-specific B and T cell responses in convalescent COVID-19 patients 6–8 months after the infection. *Med* **2**, 281-295.e4 (2021).

4. H. Marcotte, *et al.*, Immunity to SARS-CoV-2 up to 15 months after infection. *iScience* **25**, 103743 (2022).

5. F. Bertoglio, *et al.*, A SARS-CoV-2 neutralizing antibody selected from COVID-19 patients binds to the ACE2-RBD interface and is tolerant to most known RBD mutations. *Cell Rep.* **36**, 109433 (2021).

6. K. Amara, *et al.*, A Refined Protocol for Identifying Citrulline-specific Monoclonal Antibodies from Single Human B Cells from Rheumatoid Arthritis Patient Material. *BIO-Protoc.* **9,** e3347 (2019).

7. Y. Zhou, *et al.*, Enhancement versus neutralization by SARS-CoV-2 antibodies from a convalescent donor associates with distinct epitopes on the RBD. *Cell Rep.* **34**, 108699 (2021).

8. Z. Liu, *et al.*, An ultrapotent pan-β-coronavirus lineage B (β-CoV-B) neutralizing antibody locks the receptor-binding domain in closed conformation by targeting its conserved epitope. *Protein Cell* **13**, 655–675 (2021).

9. Y. Cao, *et al.*, Omicron escapes the majority of existing SARS-CoV-2 neutralizing antibodies. *Nature* **602**, 657–663 (2022).

10. S. Du, *et al.*, Structurally Resolved SARS-CoV-2 Antibody Shows High Efficacy in Severely Infected Hamsters and Provides a Potent Cocktail Pairing Strategy. *Cell* **183**, 1013-1023.e13 (2020).

11. Y. Cao, *et al.*, Potent Neutralizing Antibodies against SARS-CoV-2 Identified by High-Throughput Single-Cell Sequencing of Convalescent Patients' B Cells. *Cell* **182**, 73-84.e16 (2020).

12. Q. Yan, *et al.*, Germline IGHV3-53-encoded RBD-targeting neutralizing antibodies are commonly present in the antibody repertoires of COVID-19 patients. *Emerg. Microbes Infect.* **10**, 1097–1111.

13. J. Nie, *et al.*, Establishment and validation of a pseudovirus neutralization assay for SARS-CoV-2. *Emerg. Microbes Infect.* **9**, 680–686 (2020).

14. F. Schmidt, *et al.*, Measuring SARS-CoV-2 neutralizing antibody activity using pseudotyped and chimeric virusesSARS-CoV-2 neutralizing antibody activity. *J. Exp. Med.* **217**, e20201181 (2020).

15. E. Percivalle, *et al.*, Prevalence of SARS-CoV-2 specific neutralising antibodies in blood donors from the Lodi Red Zone in Lombardy, Italy, as at 06 April 2020. *Eurosurveillance* **25**, 2001031 (2020).

16. A. Sircar, E. T. Kim, J. J. Gray, RosettaAntibody: antibody variable region homology modeling server. *Nucleic Acids Res.* **37**, W474–W479 (2009).

17. M. Pedotti, L. Simonelli, E. Livoti, L. Varani, Computational Docking of Antibody-Antigen Complexes, Opportunities and Pitfalls Illustrated by Influenza Hemagglutinin. *Int. J. Mol. Sci.* **12**, 226–251 (2011).

18. L. Simonelli, *et al.*, Rapid Structural Characterization of Human Antibody–Antigen Complexes through Experimentally Validated Computational Docking. *J. Mol. Biol.* **396**, 1491–1507 (2010).

19. D. Van Der Spoel, *et al.*, GROMACS: Fast, flexible, and free. *J. Comput. Chem.* **26**, 1701–1718 (2005).

20. Y. Cao, *et al.*, Rational identification of potent and broad sarbecovirus-neutralizing antibody cocktails from SARS convalescents. *Cell Rep.* **41**, 11185 (2022).

21. L. Bao, *et al.*, The pathogenicity of SARS-CoV-2 in hACE2 transgenic mice. *Nature* **583**, 830–833 (2020).

22. Z. Ku, *et al.*, Nasal delivery of an IgM offers broad protection from SARS-CoV-2 variants. *Nature* **595**, 718–723 (2021).

23. Y. Cao, *et al.*, BA.2.12.1, BA.4 and BA.5 escape antibodies elicited by Omicron infection. *Nature* **608**, 593–602 (2022).

24. K. Westendorf, *et al.*, LY-CoV1404 (bebtelovimab) potently neutralizes SARS-CoV-2 variants. *Cell Rep.* **39**, 110812 (2022).

25. P. Turelli, *et al.*, P2G3 human monoclonal antibody neutralizes SARS-CoV-2 Omicron subvariants including BA.4 and BA.5 and Bebtelovimab escape mutants. bioRxiv at https://doi.org/10.1101/2022.07.28.501852 (2022).

26. J. B. Case, *et al.*, Resilience of S309 and AZD7442 monoclonal antibody treatments against infection by SARS-CoV-2 Omicron lineage strains. *Nat. Commun.* **13**, 3824 (2022).

27. A. Tuekprakhon, *et al.*, Antibody escape of SARS-CoV-2 Omicron BA.4 and BA.5 from vaccine and BA.1 serum. *Cell* **185**, 2422-2433.e13 (2022).
